# Human Genomic Diversity Where the Mediterranean Joins the Atlantic

**DOI:** 10.1093/molbev/msz288

**Published:** 2019-12-09

**Authors:** Candela L Hernández, Guillermo Pita, Bruno Cavadas, Saioa López, Luis J Sánchez-Martínez, Jean-Michel Dugoujon, Andrea Novelletto, Pedro Cuesta, Luisa Pereira, Rosario Calderón

**Affiliations:** 1 Departamento de Biodiversidad, Ecología y Evolución, Facultad de Biología, Universidad Complutense, Madrid, Spain; 2 Human Genotyping Unit-CeGen, Spanish National Cancer Research Centre (CNIO), Madrid, Spain; 3 i3S, Instituto de Investigação e Inovação em Saúde, Universidade do Porto, Porto, Portugal; 4 IPATIMUP–Instituto de Patologia e Imunologia Molecular, Universidade do Porto, Porto, Portugal; 5 UCL Cancer Institute, London, United Kingdom; 6 CNRS UMR 5288 Laboratoire d’Anthropologie Moléculaire et d’Imagerie de Synthèse (AMIS), Université Paul Sabatier Toulouse III, Toulouse, France; 7 Dipartimento di Biologia, Università Tor Vergata, Rome, Italy; 8 Centro de Proceso de Datos, Universidad Complutense, Madrid, Spain

**Keywords:** genome-wide structure, admixture, gene flow, Strait of Gibraltar, Iberia, Morocco

## Abstract

Throughout the past few years, a lively debate emerged about the timing and magnitude of the human migrations between the Iberian Peninsula and the Maghreb. Several pieces of evidence, including archaeological, anthropological, historical, and genetic data, have pointed to a complex and intermingled evolutionary history in the western Mediterranean area. To study to what extent connections across the Strait of Gibraltar and surrounding areas have shaped the present-day genomic diversity of its populations, we have performed a screening of 2.5 million single-nucleotide polymorphisms in 142 samples from southern Spain, southern Portugal, and Morocco. We built comprehensive data sets of the studied area and we implemented multistep bioinformatic approaches to assess population structure, demographic histories, and admixture dynamics. Both local and global ancestry inference showed an internal substructure in the Iberian Peninsula, mainly linked to a differential African ancestry. Western Iberia, from southern Portugal to Galicia, constituted an independent cluster within Iberia characterized by an enriched African genomic input. Migration time modeling showed recent historic dates for the admixture events occurring both in Iberia and in the North of Africa. However, an integrative vision of both paleogenomic and modern DNA data allowed us to detect chronological transitions and population turnovers that could be the result of transcontinental migrations dating back from Neolithic times. The present contribution aimed to fill the gaps in the modern human genomic record of a key geographic area, where the Mediterranean and the Atlantic come together.

## Introduction

The Iberian Peninsula is a strategic region of passage between two continents located at the western end of the Mediterranean. The entry and dispersal of anatomically modern humans from Africa into Eurasia following the “Out of Africa” model most likely occurred with the Levant as a main gateway (Lahr and Foley 1998; Mellars 2006, 2011; Stoneking and Krause 2011; Stringer 2016, among others). North Africa, excluding Egypt and part of Libya, extends to the Gulf of Gabès and contains, as an annex, the Gulf of Sirtes, forming a wide quadrilateral bordered by the Mediterranean Sea to the west, north, and east, and by the Sahara desert to the south as a major barrier. The Arabs designated the area north of Africa as “the Island of the west” (*Djezirat el Maghrib*), but its isolation refers to the global territory because it is composed of a large number of disparate regions (Gsell 1913).

The proximity between Iberia and the Maghreb not only includes the vicinity of the Strait of Gibraltar (14.4 km, its narrowest width), which has crossing difficulties due to the strong winds and dangerous currents but also the Alboran Sea (∼380 km in length and a maximum width of 180 km) to the east. It contains the small island of Alboran, as an intermediate stopover, in the distance separating Spain and Morocco, which high mountains allow the sight of land during the crossing. All these characteristics have made the western Mediterranean area most suitable for maritime contacts involving the southern and northern shores. The conjunction of archeological, anthropological, historical, and genetic data confirms the rich scenarios of ancient human movements in the region.

Migration events in the west Mediterranean were especially abundant over the Neolithic and Bronze Ages (Veeramah 2018). Then, the process continued in later prehistoric times followed by a sustained continuity during the ancient history onward. This likely picture has justified the substantial role of the Strait of Gibraltar and the Alboran Sea as the main maritime routes connecting Africa and Europe in its westernmost side.

Northern Africa has had a complex prehistory and history. Recently, new discoveries and dating of artifacts and fossils at the Jebel Irhoud Middle Stone site near Casablanca (Morocco) have reported the oldest remains of a *Homo* *sapiens* lineage in North Africa (∼310,000 ya) (Hublin et al. 2017; Richter et al. 2017). The presence of successive prehistoric industries in northern Africa associated with *H. sapiens* (Aterian, Iberomaurusian, and Capsian) has raised questions on population continuity or discontinuity (Irish 2000; Barton et al. 2013). In the Maghreb, the Iberomaurusian industry (18,000–9,500 BC) was similar to that found in Mediterranean Iberian Peninsula sites with Upper Magdalenian (13,500–11,800 BP) and Epimagdalenian (11,800–10,000 BP) chronologies. During Upper Magdalenian and until 12,800 BP, harpoons became common along Mediterranean Levantine Spanish coasts (Villaverde et al. 2012). A harpoon was found in Taforalt cave (northeastern Moroccan Rif) (Camps 1974). Evidence of big-game fishing (including harpoons) recovered from the Andalusian Nerja cave (Malaga, Spain) during the Upper Magdalenian would again support signatures of contacts between the north and southwestern Mediterranean coasts.

Southern Iberia and the Maghreb share similar characteristics with respect to the Neolithization process, a finding that has been interpreted as the consequence of strategic relationships across the western Mediterranean area (Manen et al. 2007; Linstädter et al. 2012; Isern et al. 2014; Carrasco et al. 2016; Martínez-Sánchez et al. 2018). The introduction of the Neolithic in the Iberian Peninsula (also in the Maghreb) was earlier than in other parts of Europe situated more to the east, with a rapid colonization of Iberia by Neolithic migrants (González-Fortes et al. 2019). Maritime routes were presumably the main vectors of those movements. In this respect, Cruz Berrocal (2012) and Cortés Sánchez et al. (2012) provided dates on when the Neolithic transition took place in Iberia (∼7,650–7,550 years BP) as well as sources of those migrants. Recently, Martins et al. (2015), using ^14^C dating procedures on different samples, placed the appearance of the “Neolithic package” in Iberia ∼5,500 cal. BC.

The Tartessian culture (∼800–540 BC) located in southwestern Iberia was a key component of the Iberian protohistory. Tartessos possessed important mines, which attracted people of the eastern Mediterranean and were the origin of a way, connecting the southern and northern Iberian Peninsula in its western part, which accessed important deposits of gold and tin. This way was then improved as a Roman road known as “*via de la Plata*.” During the Roman Empire, the Emperor Othon (69 BC) aggregated the province of *Mauritania Tingitana* (around northern Morocco) into the *Baetica* province (around Andalusia). In the AD 171, there was a prolonged Moorish invasion of *Baetica* and parts of *Lusitania*. In the late third century AD, Diocletian grouped *Mauritania Tingitana* together with other Iberian provinces to form the *Diocesis Hispaniarum*. Therefore, through the Roman Empire, there were recurrent human movements between the northern and southern coasts surrounding the Strait of Gibraltar, including the temporary passage of troops and tribal contingents. Muslim invasion was another period of great importance for the relationships between the Iberian Peninsula and North Africa. In the Andalusian region, the Islamic presence elapsed from the 711 to the middle of the 13th century, with the exception of its eastern part, where the *Nazari Kingdom* remained Islamic until 1492.

Over the past 5 years, a burst of interest in analyzing the Mediterranean basin both from Neolithization archeological processes (Isern et al. 2017; Guilaine 2018; Martínez-Sánchez et al. 2018) and genetic approaches (Hernández et al. 2015; Olivieri et al. 2017; Pereira et al. 2017; Sarno et al. 2017; De Angelis et al. 2018; Font-Porterias et al. 2018, among others) has unveiled important insights into its evolutionary history. The western Mediterranean represents a critical region for this kind of research. Fregel et al. (2018) detected the presence of Iberian genomic components in Late Neolithic remains from Morocco (∼3,000 BC). Genome-wide (GW) data using single-nucleotide polymorphisms (SNP) arrays in contemporary North African populations have been published by Henn et al. (2012), Botigué et al. (2013), and Arauna et al. (2017). These studies have shown high-genetic heterogeneity among North African populations and a higher representation of the Maghrebi ancestry in some Berber groups. It is specifically highlighted that the African influence on the Iberian Peninsula is, by far, more intense than in other European surrounding territories and populations.

Although the use of high-density SNP panels is highly efficient for understanding facets of the genetic and demographic histories in the Mediterranean area, the availability of genomic data with high coverage, particularly among their westernmost populations, is still scarce and more sampling and research should be performed. A particular and integrative human vision of this geographic region comprising samples from the southern Iberian Peninsula (from both Portugal and Spain) and northwestern Africa has not been explored. Therefore, in the present study, we performed a GW screening of living western Mediterranean autochthonous people from southern Portugal, southern Spain (western and eastern Andalusia), and Morocco (i.e., Berbers). Fine details on population structure, demographic histories, and admixture dynamics of this metapopulation are provided here. Comparisons with dense SNP data based on modern and ancient DNA in other European, North African and Near Eastern populations shed new insights into the timing and extent of human migrations between continents and the gene flow connecting populations.

## Results

### A Genomic Portrait across Two Continents

Our results allow us to trace a detailed view of the genomic composition of the western Mediterranean human metapopulation at the crossroads between Europe and Africa. Local ancestry inference analysis based on large-scale SNP data (1.9 million variants, see Database 1A) showed a significant (*P *<* *0.001) differential contribution of the sub-Saharan African ancestral panel among southern Iberian subpopulations (see supplementary table S1, Supplementary Material online). RFMix highlighted southern Portugal as the main Iberian target for African (using Yoruban, YRI, as the African proxy) gene input (mean value= 2.09 ± 0.71%) followed by southwestern Andalusians (Huelva) (1.21 ± 0.53%), while this influence was comparatively weaker (0.94 ± 0.28%) in Granada Andalusians. The presence and extent of African signatures in western Andalusia is in accordance with other previous data drawn from the analysis of mtDNA markers based on the same sample set (Hernández et al. 2015).

As expected, the impact of the sub-Saharan ancestral gene pool on Moroccan Berber genomes is by far stronger than that found in southern Iberians, 13.80 ± 3.41% versus 1.41 ± 0.72%, respectively. Figuig Berbers showed the highest proportion (14.02 ± 1.27%), although differences among Berber populations were not significant. Coudray et al. (2009) and Hernández et al. (2015) showed an increased representation of sub-Saharan mtDNA haplogroups in Figuig Berbers (i.e., high prevalence of L-derived lineages).

**FIg. 1.**
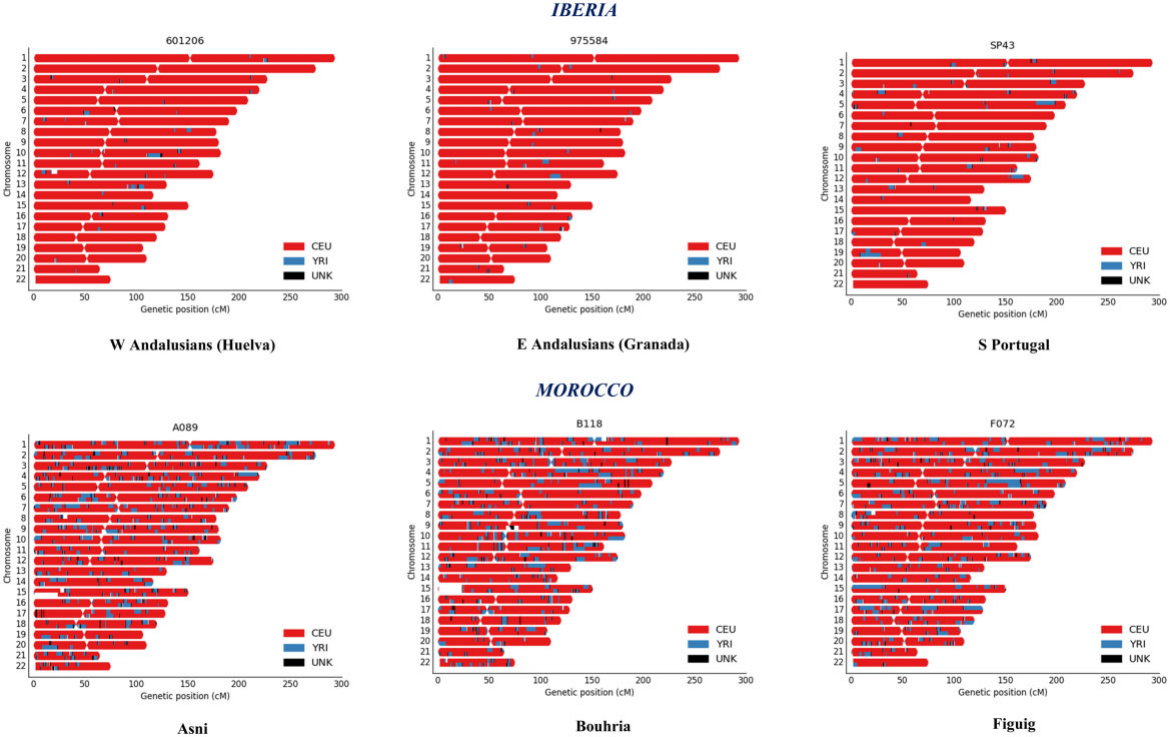
RFMix inferred karyograms in the analyzed populations from southern Iberia and northwestern Africa (Morocco). A representative individual is shown for each population harboring average values of ancestral proportions for both reference panels YRI and CEU.

Figure 1 presents the African haplotype contribution along chromosomes in representative individuals drawn from the sampled western Mediterranean individuals. The mean number of DNA segments related to sub-Saharan ancestry per sample in southern Iberians is 75.76 with an average size of 2.54 ± 3.14 cM. In Moroccan Berbers, the mean number of segments is 296.97, being the average size 3.26 ± 3.66 cM.

**FIg. 2.**
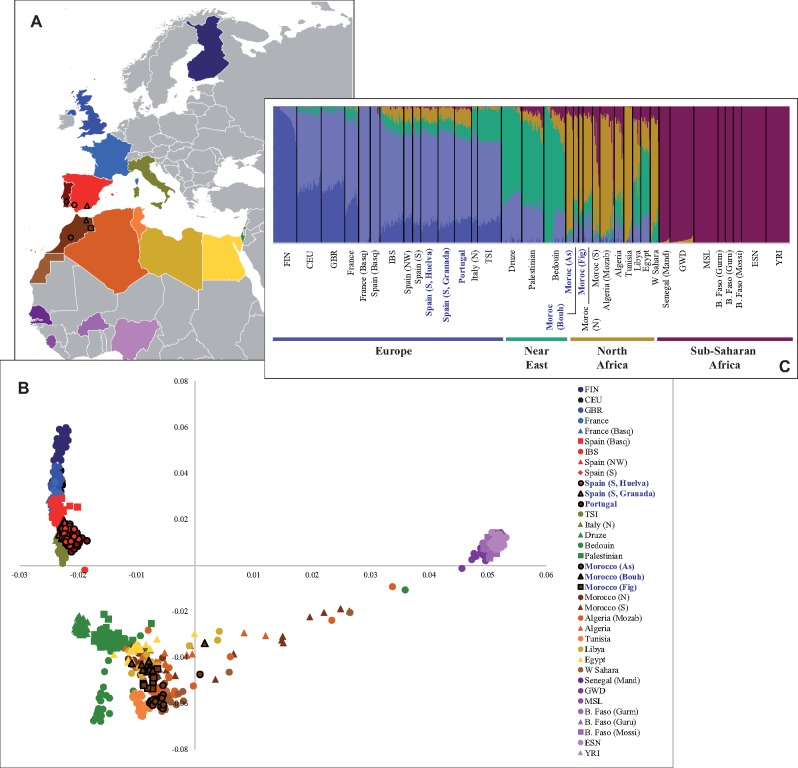
Overview of the genetic structure and global ancestry inference in a selection of European, Near Eastern, and African populations. (*A*) Countries represented in the database built for comparative analyses. The location of western Mediterranean samples studied here is highlighted in the map with symbols. (*B*) Principal components analysis (PCA). (*C*) ADMIXTURE plot for *K* = 5 ancestral clusters in the populations ordered by geographic affiliation. Both in (*B*) and (*C*), the populations genotyped in the present study are marked in bold blue face.

Moving forward into a European–Mediterranean landscape (fig. 2*A*), the principal component analysis (PCA) in figure 2*B* illustrates the structure of human populations settled along the N–S and E–W axes of the continents. The first principal component (PC1) absorbed a major variance (76.4%) and separated sub-Saharan individuals (indicated in different shades of purple) from European, Near Easterner, and North African people. Concordant with PC2 (6.9%), European samples confined in the upper left quadrant II of the plot are distributed along a latitudinal axis, with Finns at the top and Iberians plus Italians at the bottom. The N–S directionality in the genetic patterns of variation resembles the close correlation between genetic and geographic positions of populations observed elsewhere (e.g., the case of Europe, Novembre and Ramachandran 2011). Southwestern Iberian individuals (present study) appear slightly displaced from the core of the Iberian samples (excepting some Spanish Basques) toward the positive values of PC1 (quadrant IV). North African clusters are farther from one another than Europeans, whereas Near-Easterners (in green) are positioned between Europeans and North Africans. The PCA also revealed an apparent absence of a longitudinal population structure along the North African fringe. Convergent on SNP data, some North African samples display an interesting proximity to sub-Saharan people, indicating scenarios of differential admixture.

**FIg. 3.**
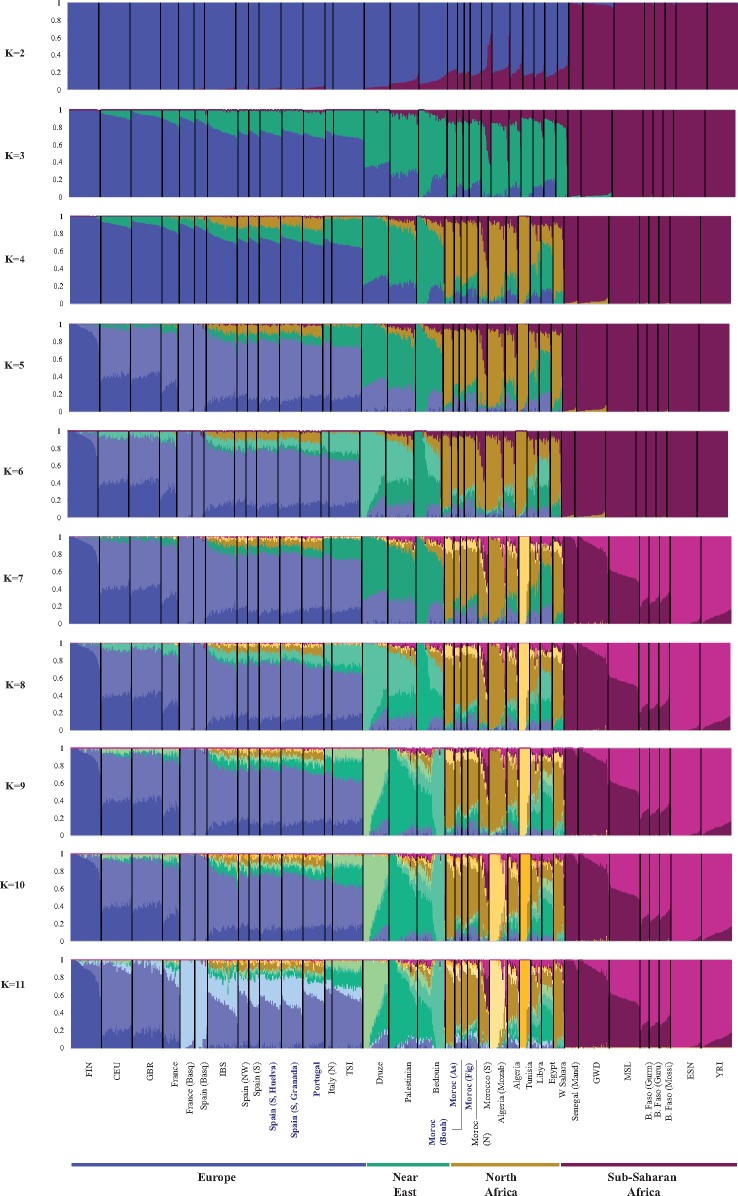
Ancestry proportions for ADMIXTURE analysis with a variable number of ancestral clusters (*K* = 2–11). See references and details on populations in supplementary table S8, Supplementary Material online. Population samples analyzed in the present study are highlighted in blue bold face. Best-fitting model is *K* = 5.

Model-based clustering analyses (ADMIXTURE) were performed at different *K* levels (2–11) (see fig. 3). The best-fitting ADMIXTURE model (minimum CV= 0.5115) contains *K* = 5 ancestral source populations (fig. 2*C*), and they are represented as “northern” and “southern” European (blue), Near Eastern (in green), North African (in ochre), and sub-Saharan (in purple) ancestries. North African people showed a variable mixture fraction of its own genome with the Near East, southern Europe, and the south of Sahara, with Berbers containing a high proportion of the North African component. Interestingly, the genetic influence of the Near East on Libyan and Egyptian genomes is noticeable. This pattern contrasts with that found in the Maghreb (western North Africa), where that influence is more reduced and comparable to that recorded from western Europe. The observed pattern seems to disagree with conclusions from Arauna et al. (2017), who stated that all of northern Africa is mixed with the Near East.

The average ancestry proportions based on ADMIXTURE analysis *K* = 5 are presented graphically in supplementary figure S1, Supplementary Material online and numerically in supplementary table S2, Supplementary Material online. Within the Iberian Peninsula, the admixture proportion of North African ancestry in southern Portugal samples was 11.17 ± 1.87%, similar to the values observed in Galicia (10.30 ± 1.64%) and western Andalusia (present study, 9.28 ± 1.79%). The Canary Islands (not selected here) exhibit extreme values of the inferred Maghrebi cluster (26%) (Guillen-Guio et al. 2018). Asni Berbers carry the highest proportion of the native Maghrebi ancestral cluster (82.74 ± 8.36%) with respect to the other Moroccan samples.

The correlation between ancestry components and geography for *K* = 5 was further explored (supplementary fig. S2, Supplementary Material online). We found a positive and significant correlation between latitude and the two European inferred ancestral clusters (Pearson coefficient= 0.652, *P *=* *3.96×10^−5^ and Pearson= 0.649, *P *=* *4.38×10^−5^, respectively), whereas the sub-Saharan global sample showed a negative strong correlation (Pearson= −0.905, *P *=* *5.25×10^−13^). By contrast, the longitude yielded a positive significant correlation (Pearson= 0.633, *P *=* *7.60×10^−5^) when considering the Near East ancestral proportion. Supplementary figure S3, Supplementary Material online, shows pairwise *F*_ST_ genetic distances estimated between populations with the heatmap depicting a clear division between sub-Saharan populations and the rest of the analyzed samples. The observed results are in close agreement with the genetic PCA findings analyzed above.

The increase in *K* ancestral sources leads to more complex patterns, suggesting the presence of microgeographic diversity in specific territories. The ADMIXTURE model for *K* = 11 yielded a CV value (0.5139) similar to that found for *K* = 5 (0.5115). The former plot permits subdividing continental groups into finer details. The European block makes clear that a specific Iberian cluster arose (in light blue color) with overrepresentation in French and Spanish Basques and a differential weight in the other Iberian populations. This cluster probably represents a signature of the descendants of people living there during the Last Glacial Maximum (LGM). Finns are fairly distinguished by a deep blue color that is almost absent in Iberians, while an intermediate blue denotes the rest of western Europeans. Non-Basque Iberians present relatively similar admixture proportions of North Africa, eastern Mediterranean’s, and Near East ancestry. The Near East appears subdivided into three groups: Druzes (in light green), Bedouins (in pale green), and Palestinians (in dark green). The genetic impact of Palestinians on the Iberian people is remarkable when compared with the other Levantine groups. According to the geographic position of Italy in the central Mediterranean, the sample analyzed here harbors a greater ancestry fraction from the Near East. In the case of North Africans, characterized by varying shades of ochre, the model permits us to distinguish Mozabite Berbers (light ochre), Tunisian Berbers (Chenini) (intermediate ochre), and Moroccan Berbers (dark ochre). The latter is the northern African component more predominant in Iberia. Sub-Saharan from western Africa and the two emerging groups (Senegalese and Nigerians) used in the present work are weakly represented in Iberia.

### Migration Events and Admixture Processes Shaping the Genomic Diversity and Population Structure in the Western Mediterranean

Next, populations were deconstructed to organize samples in genetically homogeneous clusters using a ChromoPainter–fineSTRUCTURE pipeline. The coancestry heatmap is depicted in supplementary figure S4, Supplementary Material online, and the processed tree is shown in supplementary figure S5, Supplementary Material online. The tree structure clearly correlates with the geographic adscription of individuals contained therein, as has been evidenced in previous, recent surveys (see Leslie et al. 2015; Gilbert et al. 2017). On a coarser scale and on a global perspective, four large clusters emerge: the first is defined by a deep sub-Saharan group, a second split separating Europeans of North Africans and Near Easterners, and a third branching pattern differentiating the latter two groups shape the whole tree. On a finer level of analysis (*N* = 41 clusters), western Mediterranean individuals are embedded in five North African and four Iberian clusters (see supplementary table S3 and fig. S5, Supplementary Material online). When considering Iberians as a whole and their close neighbors, three groups are detected. The first group comprises the Basques, with the C17BAS1 and C18BAS2 subclusters. The former is composed of Basques from Spain and other few Iberian non-Basque individuals, whereas the latter consists of Basques from France. Both Basque groupings appear distant from most Iberian non-Basques. Western Iberians from southern Portugal, Galicia, and other Andalusian samples compose the other Iberian cluster (C29IBE3). The remaining Iberian samples comprise the third cluster C23WAND, which gathers the bulk of western Andalusians.

Finns are differentiated from the rest of Europeans, and most Italians are grouped together, although some of them either group with some French groups or are near the western Iberian cluster (C29IBE3). British and CEU samples show a relative affinity with the Basque group. Near Eastern samples are completely differentiated from those from North Africans with the exceptions of most Egyptians and a few Libyans and Bedouins. Egyptians and Eastern Libyans are populations closer to the Near East than to the Maghreb. This could be mainly due in part to the barrier of the Libyan Desert. Most Berbers are placed in different clusters: C9TUN, C15MOZ, and C10MOR, probably reflecting a clear Berber population structure associated with the old kingdoms and contemporary nations in the region.

**FIg. 4.**
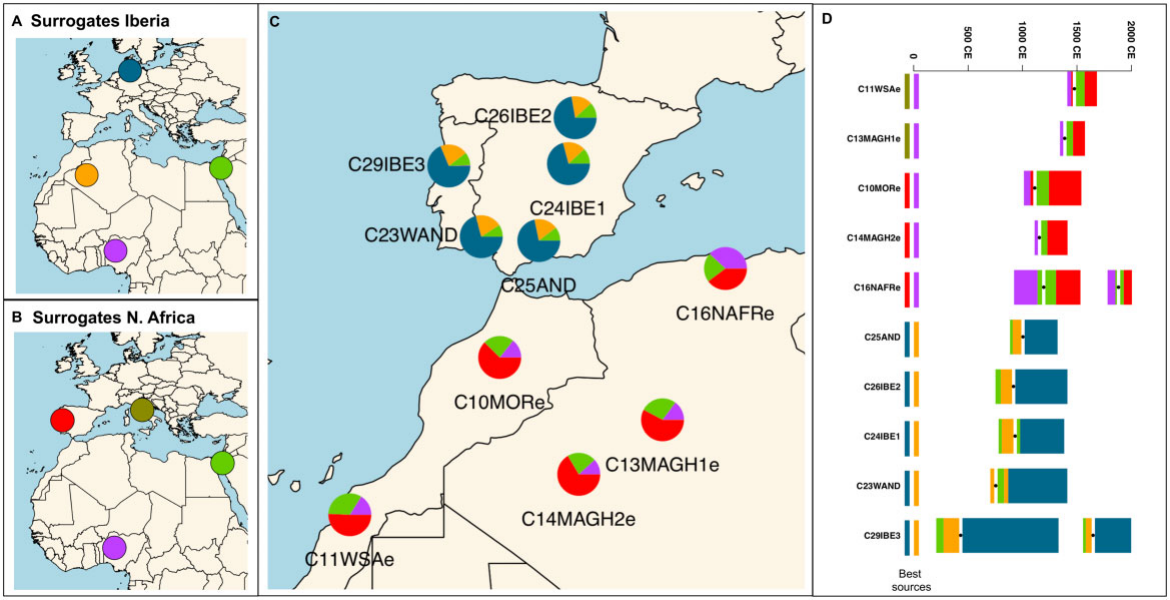
GLOBETROTTER results. (*A*) Surrogates for the Iberian recipient clusters. (*B*) Surrogates for the North African recipient clusters. (*C*) Admixture proportions inferred from a nonnegative least squares approach. (*D*) GLOBETROTTER results for each cluster. Black points show the mean admixture dates (calculated as 2,000−25×generation time). Barplot colors show the source populations that contributed to the admixture (see *A* and *B*). Bar width represents the bootstrap 100% CI. Best sources are indicated in the left.

Clusters built with fineSTRUCTURE were subsequently used for reconstructing past admixture events using GLOBETROTTER and the results are presented in figure 4 and supplementary table S4, Supplementary Material online. When considering North Africans as recipients, the most probable donors were Iberian and Italian Peninsulas. In order to minimize an underestimation of inferred European contribution due to some Iberian regions having in themselves North African input, we tested several Iberian (C23WAND/C24IBE1/C25AND/C29IBE3) and one Italian (C22ITA1) donor clusters to explore the dichotomy between these two European sources into North Africa (see supplementary table S4*A* and *B*, Supplementary Material online).

Admixture events inferred were defined by two population sources (1 and 2) with different contributions, the best proxy of each source and admixture dates. Most of the estimated admixture episodes for both the Iberian and North African recipient clusters were unique (one-date admixture events). GLOBETROTTER detected two additional admixture pulses for the most diverse clusters on the data set as a reflection of their complex history.

Point estimates of dates spanned from ∼54 to 4 generations ago, that is, from the 7th to 19th centuries CE (see supplementary table S4, Supplementary Material online). Previous evidence also points to historic migratory events to explain admixture processes in both Iberia and North Africa. Accordingly, Moorjani et al. (2011) estimated dates of a sub-Saharan African admixing source in Portugal and Spain by ∼45 ± 5 and 55 ± 3 generations ago, respectively. Recently, Bycroft et al. (2019) dated admixture events involving European and northwest African source groups to ∼45–35 generations ago (860–1,120 CE). In addition, analyses for North African populations as recipients highlight the effects of recent historical migratory movements, and dates of admixture were mainly explained by the Islamic expansion in Iberia (7th century CE) and the trans-Atlantic/trans-Saharan slave trade (17th century CE) (Arauna et al. 2017).

With respect to Iberian recipients, virtually all cases in the present study included an admixture event between a major European-like source (82.3 ± 6.5% as an average) and an African source. The latter source is best represented by a North African population and not by a sub-Saharan population. Since we set two different scenarios, C10MOR or C14MAGH2, as North African donors, it should be interesting to search for potential differences along the five Iberian recipient clusters considered (C23WAND, C24IBE1, C25AND, C26IBE2, and C29IBE3). When first using C10MOR as the proxy for North African ancestry, this component is displaced as best-source 1 in two cases (see C24IBE1b and C26IBE2b, supplementary table S4*A*, Supplementary Material online); if considering C14MAGH2 instead, the African contribution is present in the rest of comparisons. Therefore, the North African source would have a greater relative proportion regarding the European source when using C14MAGH2 versus C10MOR (mean values: 20.0 ± 5.9% and 14.2 ± 5.3%, respectively). The former cluster comprises northern Moroccan samples and three Algerian individuals, whereas the latter is restricted to Morocco. As stated before, GLOBETROTTER inferred two admixture pulses for western Iberian cluster C29IBE3 (see supplementary table S4*B*, Supplementary Material online) encompassing date estimates of 890 CE.

As shown in supplementary table S4*A* and *B*, Supplementary Material online, for the Iberian recipient clusters, source 2 remained constant in all cases (i.e., European source, C21NEUR), whereas the best-source 1 representative varied between the two North African donors distinguished by cluster compositions. For North African receptors, however, the donor clusters are recurrently represented by Yoruban (C41YRI cluster) as the best source; the other source would be Mediterranean Europe. It is interesting to note that when using the western Iberia C29IBE3 cluster (comprising individuals with family origins in western Iberia, see scenarios marked by suffix “e”), it replaces the Italian cluster (C22ITA1) (see supplementary table S4*A*, Supplementary Material online). When given further support by considering C29IBE3 as the most likely Iberian donor, the relative proportion of the European source (when compared with the African donor) reaches the maximum value (mean European proportion in scenarios: A, 78.8 ± 4.6%; C, 75.5 ± 6.6%; D, 81 ± 5.0%; and E, 82 ± 3.3%).

The cluster C16NAFR unveiled a higher complexity mainly due to two inferred admixture episodes. As for single-pulse events, the introduction of the Iberian C29IBE3 cluster as a donor (C16NAFRe test, supplementary table S4*B*, Supplementary Material online) resulted in the best-guess proxy of source 2. Nevertheless, deeper migration events in the region detected through uniparental markers (see Discussion) would be masked by the autosomal variation as a methodological consequence of the recombination (Fernandes et al. 2015). According to Hellenthal et al. (2014), admixture dates provide higher bounds to migration dates because migrations occurred before population admixtures and after mixing, and temporal dates would estimate a time posterior to the beginning of the mixing.

### Evidence Drawn from Ancient and Modern Genomes

**FIg. 5.**
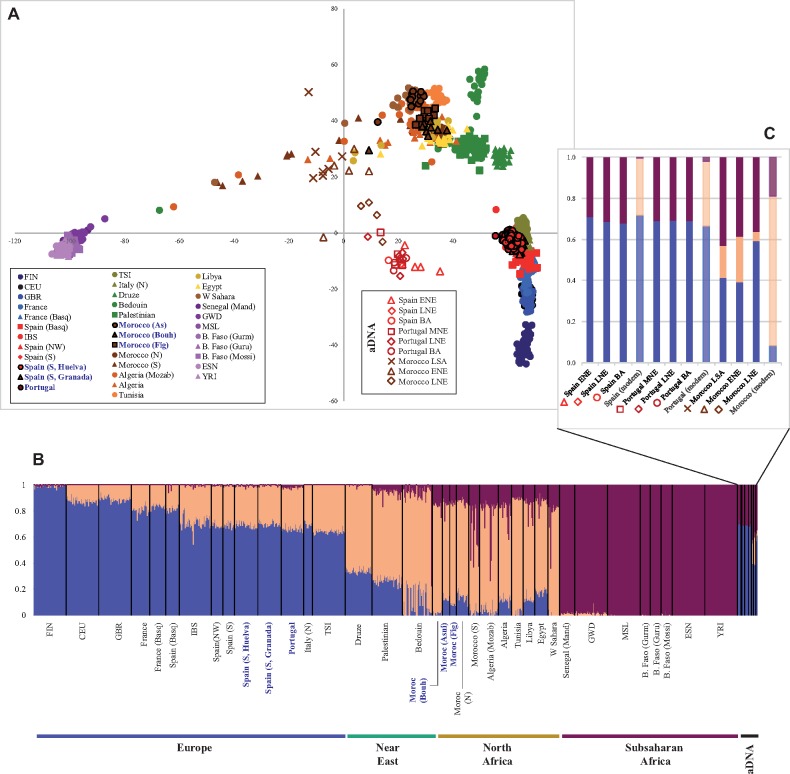
A genomic chronology of the studied area. Ancient DNA samples are integrated with Database 2B. (*A*) PCA built with ancient and modern samples (see supplementary tables S8 and S10, Supplementary Material online). (*B*) ADMIXTURE plot (*K* = 3). (*C*) Mean global ancestry proportions (*K* = 3) estimated in Spanish, Portuguese, and Moroccan periods (LSA, Late Stone Age; ENE, Early Neolithic; MNE, Middle Neolithic; LNE, Late Neolithic; BA, Bronze Age). Average values for modern samples (see pale bars) were calculated from Database 2B (supplementary table S8, Supplementary Material online) including the following populations: IBS, SPGA, SPBA, SPAN, SPWA, SPEA (Spain modern), SPOR (Portugal modern), and MAAS, MABO, MAFI, MANO, MASO (Morocco modern).

Finally, given the current boom in aDNA data for the western Mediterranean area, we integrated our present genomic evidence in a temporal context. The PCA composed of both aDNA assembled genomic data and the modern data set show that aDNA samples are located halfway between the European-Near Eastern-North African gradient and the sub-Saharan cluster, being distributed in a sort of diagonal axis (see fig. 5*A*). Ancient samples display considerable distances among them, in particular Moroccan Taforalt (LSA, brown crosses) and El Toro, Antequera, Andalusia Early Neolithic (ENE, red triangles).

For Moroccan aDNA samples, a chronological cline is evident, but for the Iberian samples, it would be more challenging to detect this tendency. Both groups of ancient samples deviate slightly from the corresponding present ones toward the sub-Saharan cluster. The ADMIXTURE results can shed some light on the temporal variations of different sources of ancestry in western Mediterranean over time. In figure 5*C*, we can see that the percentage of the blue European component is similar in ancient and present-day European samples (0.67–0.72), while this component, high in ancient Moroccan samples (0.39–0.59), is reduced sharply in present-day samples (0.08). The ochre component (“Mediterranean” that could represent a Maghrebi/Near Eastern ancestry) changes from (0.05–0.22) in ancient Moroccan samples to 0.73 in contemporaneous ones, while the corresponding change in Iberians goes from zero to (0.28–0.32). The purple sub-Saharan component moves from (0.36–0.43) to 0.19 in Moroccans and from (0.29–0.32) to (0.01–0.02) in Iberians. In short, the pass in the region from ancient to present-day populations consists for Iberia in the substitution of the sub-Saharan component by that “Mediterranean” cluster, while in Moroccans its considerable rise is compensated with notable reductions of European and sub-Saharan ones. These results suggest that the Maghreb component was mostly transferred to Iberia after the Neolithic, obscuring previous genetic relationships between Iberia and North Africa.

## Discussion

The Iberian Peninsula and northern Africa could be considered subcontinents of Europe and Africa, being separated in their geographic framework by physical barriers, the Pyrenees and the Sahara desert, respectively, and “connected” to each other by water masses, mainly the Mediterranean Sea. These features have determined particular demographic and migratory processes experienced by their populations and, hence, their present-day genomic profiles.

Here, we have provided an integrative vision of the genomic profile and population dynamics of the western Mediterranean, not only circumscribed to Spain and Morocco but also considering the Atlantic edge by including Portugal. Our results have shown that this metapopulation, as a set of subpopulations connected by migration (Hanski and Gilpin 1991), represents a clear genetic crossroads between Europe and Africa and a classic example for exploring human evolutionary histories across continents.

Large-scale human movements have likely implied maritime travel as a more effective strategy than land routes. In this context, the Mediterranean Sea should be understood as a transport surface (Braudel 1987) that favored the connectivity among its regions. That attributed “role” was especially active from Neolithic times on, just when it started to behave as a highway for the lateral transmission of textiles, metals, technologies, and people (Horden and Purcell 2000; Broodbank 2006). The Near Eastern ancestry was found in the opposite extreme of the Mediterranean basin (i.e., the Iberian Peninsula) and would be a reflection of these long-distance migrations.

According to our GW data, Iberians, including southern autochthonous people, are mainly mixing outcomes of ancient settlers in the Peninsula, represented by the Basques and northwestern Europeans, with a reduced contribution of Maghreb and Near East as well as a minor component of sub-Saharan people. The latter influence seems to be old because of the few and short DNA tracts observed on Iberian chromosomes. By contrast, the comparatively abundant, larger sub-Saharan tracts detected on Moroccan Berber chromosomes reveal that most admixtures should be relatively recent, probably after the domestication of camels, which initiated, around the fifth century CE, the trans-Saharan caravan travels connecting the Niger basin to the North African fringe (Harich et al. 2010; Fortes-Lima et al. 2014). In this context, GW data analyses have shown that the sub-Saharan component in the Maghreb is coherently much stronger in its more southern enclaves.

In the ADMIXTURE analysis performed in this study, Basques were almost entirely composed of a single cluster, which could be closely similar to the LGM Iberian hunter-gatherers. The demographic history of the Franco-Cantabrian region and the results of the analyses performed here, which show very low admixture of Basques with other populations, in particular, with Near Easterners, provide some support to this hypothesis. This geographic region, where Basques were settled, was profusely populated during the LGM and this dynamic was sustained later. After that period, hunter-gatherers living in the Franco-Iberian refuge expanded to other parts of Europe, which were, at that time, almost uninhabited (Bocquet-Appel et al. 2005). Nevertheless, the idea of Basques as descendant of LGM hunter-gatherers with no input from following migration has been refuted by recent aDNA studies (Günther et al. 2015; Valdiosera et al. 2018; Olalde et al. 2019). Several ancient DNA studies show that Basques are like other Iberian populations admixed with Neolithic coming from the Middle East and further received input from the Bronze Age Steppe populations. It could be that these old admixtures events (with Near Eastern and Steppes origins) have been erased by following drift. Because of the considerable increase in the North Africa component in the Maghreb after the Neolithic, most Iberian admixture events would have taken place later than that period. However, by applying the GLOBETROTTER methodology, which uses high-density SNP coverage, the date estimates for admixture events across western Mediterranean in the present study are clearly restricted to historic times. The properties of recombination result in the inability of dense SNP screenings for properly date deep migration episodes, reaching an upper limit of around 4,500 BP (∼160 generations), after which recombination removes reliable detectable haplotypes (Hellenthal et al. 2014). The stochastic process of recombination is extremely complicated, with some pieces of the “ancestral recombination graph” showing their MRCA long before others (Nordborg 2003). Consequently, estimations of admixture dates are considerably young and uncertain.

The long history of extensive trans-Mediterranean gene flow between the Iberian Peninsula and northern Africa has been well established, and contrasted evidence is supported from different sources of information. An example can be found in the relatively high African genetic signatures in Iberia, especially along its western side. These signals have been mainly detected through haploid, uniparental markers. Modern mtDNA studies have revealed that a certain proportion of African lineages were introduced in the Iberian Peninsula around Early Holocenic times (Cerezo et al. 2012; Hernández et al. 2015). Reciprocally, the input of some European maternal lineages—via Iberia—into North Africa was restricted again to prehistoric times (Cherni et al. 2009; Ottoni et al. 2010; Hernández et al. 2017). Similarly, analyses of ancient mtDNA samples are also very enlightening. Recently, González-Fortes et al. (2019) reported a mitogenome of clear sub-Saharan origin (L2a1) in a 3,600-year-old sample from an Andalusian cave (Córdoba), suggesting transcontinental migrations predating the Bronze Age. Additionally, in Camino de las Yeseras, an especially interesting Late Chalcolithic central Iberian site, a female individual was recovered and genetically distinguished by harboring the L1b mtDNA lineage (Szécsényi-Nagy et al. 2017). Both maternal haplogroups are frequently found in western Africa (see Hernández et al. 2015 and data sets S3 and S4 therein). Olalde et al. (2019) reported a North African ancestry for both mtDNA (M1a1b1) and Y-chromosome (E1b1b1a) lineages in a male individual from Camino de las Yeseras.

The shape of North Africa, with a remarkable extension in the longitudinal direction and latitudinal shortness, has greatly contributed to its distinguished interpopulation genetic diversity landscape. For example, Alexandria (Egypt) and Tunis (Tunisia) are 4,500 km apart, and within Libya, the broad desert territory between Tripoli and Benghazi, has been scarcely populated from antiquity to recent times. Likewise, Berbers in the Maghreb are genetically differentiated according to ethnic origins (i.e., Morocco, Algeria, Tunisia) indicating a prolonged isolation among them. The European component present in Berbers, at least those settled in northwestern Africa (present study), is generally higher than that observed from the Near East, signifying more intense contacts with the west than eastern Mediterranean. A portion of that European component comes from the Basque genome, indicating old relationships with Iberia. Unfortunately, with data emerging from the present study, it is not possible to discern whether most of the Near East ancestry component in Berber genomes is a result of Islamic expansion or due to more ancient times.

Some genetic diversity studies have observed closer relationships between North Africans and Europeans than the former with sub-Saharans (Ennafaa et al. 2009; Fadhlaoui-Zid et al. 2013). However, interesting and different scenarios have been found that depend on the nature of genetic markers of study. For instance, the European ancestry inferred for North African individuals appears less represented when using GW data than when using haploid markers. This is especially evident for mtDNA, where the European contribution can exceed 50% (Coudray et al. 2009; Font-Porterias et al. 2018). Instead, genomic analyses performed here showed an average European ancestral proportion in northern Africa of 12.2 ± 7.1% (see supplementary table S2 and fig. S1, Supplementary Material online). Nevertheless, and as expected, the major proportion of North African genomic diversity, such as ADMIXTURE data states, would be due to the “native” Maghrebi cluster (62.5 ± 20.5%), which is far more frequent in the west than in its eastern side (see fig. 2*C*). In fact, the weight of the Maghrebi component is relatively low in Libya and Egypt, where high rates of Near East ancestry are observed. This interesting finding confirms the crucial role of the Libyan desert as a physical barrier to human mobility. The presence of Near Eastern ancestry, which follows an opposite distribution with a gradient toward the East, has been linked to the Arabian expansion (Hollfelder et al. 2017).

The Iberian samples in this study do not record a sharp population turnover until more recent times (from the Bronze Age, BA to present times) at least for the basal situation of three ancestries tested (see fig. 5*C*). The impact of Bronze Age Steppe herders (Yamnaya people) is thought to have been massive in central and northern European populations (Allentoft et al. 2015; Haak et al. 2015). However, the relevance of this demographic expansion in southwestern Europe is under debate. Ancient DNA studies have shown that the Neolithic transition caused major changes in the Iberian gene pool, whereas the Yamnaya input had a limited effect (Martiniano et al. 2017; Szécsényi-Nagy et al. 2017; Valdiosera et al. 2018). This idea has been challenged very recently, suggesting that ∼40% of the Iberian’s ancestry was replaced by Steppe people by 2,000 BCE (Olalde et al. 2019). In the present study, we observed a switch of ∼30% of the Iberian ancestry from sub-Saharan into another component, called “Mediterranean” for its population distribution, after BA (see fig. 5*C*). This fact could chronologically match the impact of BA migrations. However, it would be puzzling to associate the “Mediterranean” contributor to the Steppe input, since modern Moroccan samples are mostly characterized by this ancestral source. Another possible interpretation could lie in the definition of this ancestry because of gene flow processes across the Mediterranean basin, encompassing both Near Eastern and North African sources, which occurred due to the development of extensive seafaring activity across the Mediterranean basin in the last millennia. To understand the BA-to-modern Iberian transition, we can further explore here the definition of the western Iberian cluster-C29IBE3 and its likely correspondence, both for admixture time estimation (the deeper event recorded of all scenarios tested) and for geographic distribution, with the historical connection between SW and NW Iberia by means of “*via de la Plata*” Roman road. Our genomic data reflect that this road, that enabled military and commercial activities, could have acted as a vehicle for the spread of (African) genes, and that have significantly defined the contemporary internal structure within the Iberian Peninsula (see Fortes-Lima et al. 2014; Hernández et al. 2014).

Although current available methods for dating admixture events from GW data are likely biased toward more recent episodes (Pagani and Crevecoeur 2019), global ancestry procedures allow the observation of traces of chronological transitions and old events across western Mediterranean. Therefore, we must open the time boundaries for south to north (and reciprocally) population movements between Iberia and the Maghreb far deeper than the medieval Muslim conquest and the Roman period.

In conclusion, the great complexity of the analyzed area regarding geographic and climatic conditions, demographic history, and archeological record has shaped the genomic diversity of their contemporary populations. Western Iberia—its southernmost part in particular—is the European hotspot regarding African genomic contribution. The Maghreb harbors several ancestry sources enriched by an Iberian influx dating back from Neolithic times. Broadly, the geographic link-up of Iberia and the Maghreb should be considered a transition area between two continents, acting as a bridge for genetic exchanges. Although GW studies have the potential to provide an accurate overview of the human past, including population origins, migration and admixture events, and demographic history of populations, haploid marker analysis should still be explored for tracing deep structure and relationships between populations. The present contribution aimed to fill the gaps in the modern human genomic record of a key geographic area, where the Mediterranean and the Atlantic come together.

## Materials and Methods

### Populations and Sample Selection

In the present work, we performed a GW screening of six western Mediterranean populations. The southern Iberian sample comprises three autochthonous populations, one from Portugal, a national territory separated from Spain by a border from medieval times, and two others from the western and eastern sides of the Spanish Andalusian region, from Huelva and Granada provinces, respectively, and Moroccan Berber native people from Asni, Bouhria, and Figuig representing the three North African samples. Individuals analyzed here were unrelated, and samples are the result of a random selection from their respective source population sample sets. Each participant provided written informed consent as well as information on familiar geographic origins.

Biological samples from both southern Iberia and Morocco analyzed in the present study have been previously used in other genetic diversity studies based on uniparental and autosomal markers (Pereira et al. 2005; Coudray et al. 2009; Ambrosio et al. 2010; Hernández et al. 2014). Details on the geographic, demographic, historic, and genetic background of these populations can be found in the abovementioned references.

### Genotyping and Curation of SNPs

A total of 142 western Mediterranean individuals (74 males, 68 females, see supplementary table S5, Supplementary Material online) were genotyped with Illumina’s Omni2.5-8 BeadChip array at the Spanish National Cancer Research Centre (CNIO, *Centro Nacional de Investigaciones Oncológicas*) in the Human Genotyping laboratory (CeGen). The raw data genotyped consisted of 2,372,784 SNP markers with a mean genotyping rate of 99.5%. A duplicated sample was used as an internal control (HapMap code Na12775) with a reproducibility of >99.9%. The genotypes from the individuals analyzed in this study can be accessed from the EGA repository (European Genome-Phenome Archive; Study Accession Number: EGAS00001003901). Mitochondrial DNA (mtDNA) haplogroups were inferred from chip data by using Haplogrep (Weissensteiner et al. 2016). The resulting maternal profile of the Iberian and Moroccan samples used here (see supplementary table S6, Supplementary Material online) closely agreed with that published in earlier surveys (Pereira et al. 2000, 2005; Coudray et al. 2009; Hernández et al. 2014, 2015, 2017).

A systematic workflow for quality control and genomic sample set assembly was performed, and a schematic view is displayed in supplementary figure S6, Supplementary Material online. The hg19/GRCh37 human reference sequence was used in all analyses. Curation of SNPs was undertaken using PLINK v.1.9 (Chang et al. 2015) at every step of the pipeline. Criteria for variant pruning were as follows: missing genotype rate per person and per SNP (both >5%), significant departure (10^−7^) from Hardy–Weinberg equilibrium, and a minor allele frequency threshold of 1%. Recent genetic relatedness was excluded by applying the PC-Relate approach (Conomos et al. 2016).

The resulting sample set studied in the present survey was merged with nine populations from the 1000 Genomes Project (1000GP) phase 3 (Auton et al. 2015) to obtain a high-resolution database comprising 1.87 million SNPs (Database 1A: see supplementary table S7, Supplementary Material online, for details on the populations included). The population genomic information taken from the literature was selected following criteria of reproducibility of array resolution and interest of the geographic area as well. Afterward, we increased the sample set by adding 21 populations mainly from southwestern Europe, North Africa, and Near East to obtain a broader picture of the Mediterranean genomic diversity. Positions were lifted to build 37 when necessary by using liftOver (https://genome.ucsc.edu/util.html). Data from the Illumina and Affymetrix platforms were merged on physical position. The final merged data set (Database 2A) resulted in 84,586 SNPs for 1,523 individuals across 36 present-day populations. Details on this database are shown in supplementary table S8, Supplementary Material online, and the geographic location of all populations considered in the current study is depicted in supplementary figure S7, Supplementary Material online. Markers contained in both high- and low-resolution approaches were pruned for linkage disequilibrium (LD, *r*^2^> 0.2) to obtain 465,598 (Database 1B) and 63,189 (Database 2B) SNP sets, respectively. Each database was used for different methodological procedures (the general bioinformatic pipeline is depicted in supplementary fig. S8, Supplementary Material online).

### Ancestry Patterns and Population Structure Analysis

Database 1A was first phased with SHAPEIT2 (Delaneau et al. 2013). Then, we assessed local ancestry inference by means of RFMix v1.5.4 (Maples et al. 2013), using two steps of the expectation-maximization (EM) algorithm. This method aims to infer the ancestry of each segment of the genome as a mixture of two or more parental panels of haplotypes. The reference panels used were CEU (northern European ancestry) and YRI (Yoruba Nigerian African ancestry) from the 1000GP. Average ancestry proportions were analyzed, and differences between populations were tested by Student’s *t*-test with the Welch correction when the variances between the pairs of groups differed significantly.

The LD-pruned high-resolution database (Database 1B) was used to check for possible outliers or errors in data merging by means of an exploratory PCA using the *smartpca* program in the EIGENSOFT package (Patterson et al. 2006) (data not shown).

A geographically broader picture of ancestry inference was then assessed from Database 2B (LD-pruned low-resolution data). The RFMix approach, as indicated above, was complemented with the development of a model-based estimation of global ancestry using ADMIXTURE (Alexander et al. 2009). Models were explored by testing different numbers of ancestral populations on separate runs (from *K* = 2 to *K* = 13 clusters). A balanced sample size was used across populations. A cross-validation (CV) test was performed to validate the best-fitting estimation (*K* = 5, CV error= 0.5115, see supplementary fig. S9*A*, Supplementary Material online). The selection of *K* = 5 was based merely on the CV values. It is true that these values are very close from *K* = 4 to *K* = 6, and that *K* = 4 could be more parsimonious, but in *K* = 5 we can observe the split between two inferred European sources. To reinforce the interpretations based on ADMIXTURE, we implemented the badMIXTURE approach (Lawson et al. 2018). In general, we observe small residuals, being the higher ones concentrated in sub-Saharans and Near Easterners (see supplementary fig. S10, Supplementary Material online). Consequently, the results provided by ADMIXTURE constitute robust inferences of the ancestries.

Database 2B was also used to evaluate population structure by means of PCA (Patterson et al. 2006) and the calculation of *F*_ST_ (a measure of genetic differentiation) between pairs of populations.

### Admixture Events and Demographic Inferences

Migration timing modeling was performed by using a haplotype-based methodology. Specifically, we used a *chromosome painting* multistep approach for inferring ancestry blocks (“chunks”), and it allowed us to classify individuals into genetically homogeneous clusters and to sequentially date evolutionary events. First, we developed an “all vs. all” analysis for all samples included in our extended data set (Database 2A) considering the 1,523 individuals both as donors and recipients in ChromoPainter v2 (Lawson et al. 2012). The *Mut* (mutation rate) and *Ne* (switch rate) parameters were estimated by an initial analysis with ten EM steps in four chromosomes (1, 4, 15, and 22), and the values were then incorporated into the program to perform analysis with all chromosomes. The resulting matrices showed the amount (counts) and size (lengths) of DNA copied from each donor individual by each recipient individual per chromosome. Data were combined into single files by using CHROMOCOMBINE, and the normalization parameter “*c*” (effective number of chunks or independent genetic elements) was estimated as well.

The combined coancestry matrix was used for fineSTRUCTURE analysis (Lawson et al. 2012) by performing two million Markov-Chain-Monte-Carlo (MCMC) iterations, with a “burn-in” of one million, and sampling from the posterior distribution every 10,000 iterations after the “burn-in” effect. This process was followed by tree estimation performing 100,000 additional hill-climbing steps. We obtained three versions of the tree using different random seeds that reached similar population branch structures.

Individuals were grouped into 119 clusters. We performed a manual exploration of the tree to define genetically homogeneous groups by considering a minimum size of ten individuals per cluster. The 41 clusters finally established showed a fair correlation with the geographic affiliation of populations included (see the composition of each cluster in supplementary table S3, Supplementary Material online). In this stage, we considered the clusters set as “metapopulations” to test a series of admixture and population migration scenarios using ChromoPainter, selecting “target” (to detect admixture in) and “surrogate” (to define the ancestral DNA involved in the admixture episode). As done for previous methods, we proposed different tests for Europe and North African individuals. For the Iberian clusters (C23–C26 and C29), we defined four putative ancestral sources represented by surrogate clusters: Near East (C4), North Africa (C14), Europe (C21), and sub-Saharan Africa (C41). For the selection of these donor clusters, we considered both the size of the group and its population composition. To determine the dependence of the results on the selected surrogates, we performed an additional test replacing C14 with C10 as a source of North African ancestry. In North African target clusters (C10, C11, C13, C14, and C16) we determined the following initial ancestral source groups: Near East (C14), central Mediterranean Europe (C22), western Mediterranean Europe (C25), and sub-Saharan Africa (C41). Likewise, we developed analogous tests replacing the Iberian surrogate C25 with by C23, C24, and C29 in independent runs (supplementary table S9, Supplementary Material online, records all scenarios analyzed).

For the new ChromoPainter runs, we again estimated the *Mut* and *Ne* parameters. The combined coancestry matrices for each scenario (Iberia A–B, North Africa A, C, D, E) were first explored to analyze the proportion of the genome copied from each surrogate cluster by each target cluster. Both the total length of genomic material copied from donors and the total number of haplotype segments were standardized to sum up 100% in each recipient cluster.

Then, ChromoPainter *outputs* were used in GLOBETROTTER to identify and date recent admixture events in the Iberian and North African clusters using the painting profiles and surrogates defined for each scenario (Hellenthal et al. 2014). We used the default LD decay curve range of 1–50 cM and bin size of 0.1 cM when considering the distance between genome segments and five iterations of GLOBETROTTER’s alternating source composition and inference of the admixture date. Next, 95% confident intervals around the point estimates of the admixture date for each event were inferred by 100 bootstrap resamplings. To account for spurious signals of LD not attributable to admixture, all scenarios were run with the options null.ind: 1 and null.ind: 0 (Hellenthal et al. 2014). As the results were largely consistent, we only report results for null.ind: 1.

### A Chronological Framework for the Western Mediterranean

Relevant genome diversity studies on ancient DNA samples on Iberia and Morocco (Fregel et al. 2018; van de Loosdrecht et al. 2018) provide a contextualizing portrayal for ancient transcontinental contacts and ancestral inference for these populations. To gain further insights into gene flow processes across continents, we incorporated those aDNA samples together with other pertinent individuals from the same geographic area (Martiniano et al. 2017; Valdiosera et al. 2018) into Database 2B. These available paleogenomic data permit additional population structure analyses and global ancestry inference. Supplementary table S10, Supplementary Material online, shows details on aDNA selected samples belonging to Spain, Portugal, and Morocco and spanning from the Moroccan Late Stone Age (Taforalt cave) to the Spanish Bronze Age.

We implemented two strategies for merging aDNA individuals with our modern sample sets following Fregel et al. (2018). First, we used the LASER method (Wang et al. 2015) to estimate ancestry by using low-coverage sequence reads with a Procrustes projection into a PCA space. Then, we built another PCA with *smartpca* program in EIGENSOFT (Patterson et al. 2006) with the *lsqproject* option selected, a feature implemented to correct for missing genotyping data. Both multivariate plots depicted a similar topology of aDNA samples. Likewise, we used the *lsqproject* approach to perform an ADMIXTURE analysis with the complete sample set including modern and aDNA samples. Global ancestry analysis was tested from *K* = 2 to *K* = 10 ancestral populations. Supplementary figure S9*B*, Supplementary Material online, shows the CV values for each test. We selected *K* = 3 for interpretations as it represents the sharpest decrease in error. In addition, from *K* = 4–10, an independent ancestral cluster is inferred as a single contributor in all aDNA samples (data not shown), making it difficult at these *K*-tests to envision genomic relationships between ancient and modern samples.

## Supplementary Material

msz288-Supplementary_Data
