# Supplementary material for: Human Genomic Diversity Where the Mediterranean Joins the Atlantic
Source: Mol Biol Evol. 2019 Dec 9;37(4):1041–55. doi: 10.1093/molbev/msz288 (PMC7086172; doi:10.1093/molbev/msz288)
Supplement: msz288-Supplementary_Data [file msz288-supplementary_data.zip › msz288-Suppl_Data/MBE-19-0603_Supp Figs_R2.pdf]

Supplementary Figures

Human genomic diversity where the Mediterranean joins the Atlantic

Candela L. Hernández, Guillermo Pita, Bruno Cavadas, Saioa López, Luis J. Sánchez-Martínez, Jean-Michel Dugoujon, Andrea Novelletto, Pedro Cuesta, Luisa Pereira & Rosario Calderón

Figure S1. Mean global ancestry proportions (ADMIXTURE K=5) 2

Figure S2. Correlation tests ancestry proportions vs. geography 3

Figure S3. F_{ST} heatmap 4

Figure S4. fineSTRUCTURE coancestry heatmap 5

Figure S5. fineSTRUCTURE tree 6

Figure S6. SNP curation and database merging 7

Figure S7. The geography of population distribution 8

Figure S8. Bioinformatic pipeline 9

Figure S9. Cross-validation test (ADMIXTURE) 10

Figure S10. badMIXTURE analysis 11

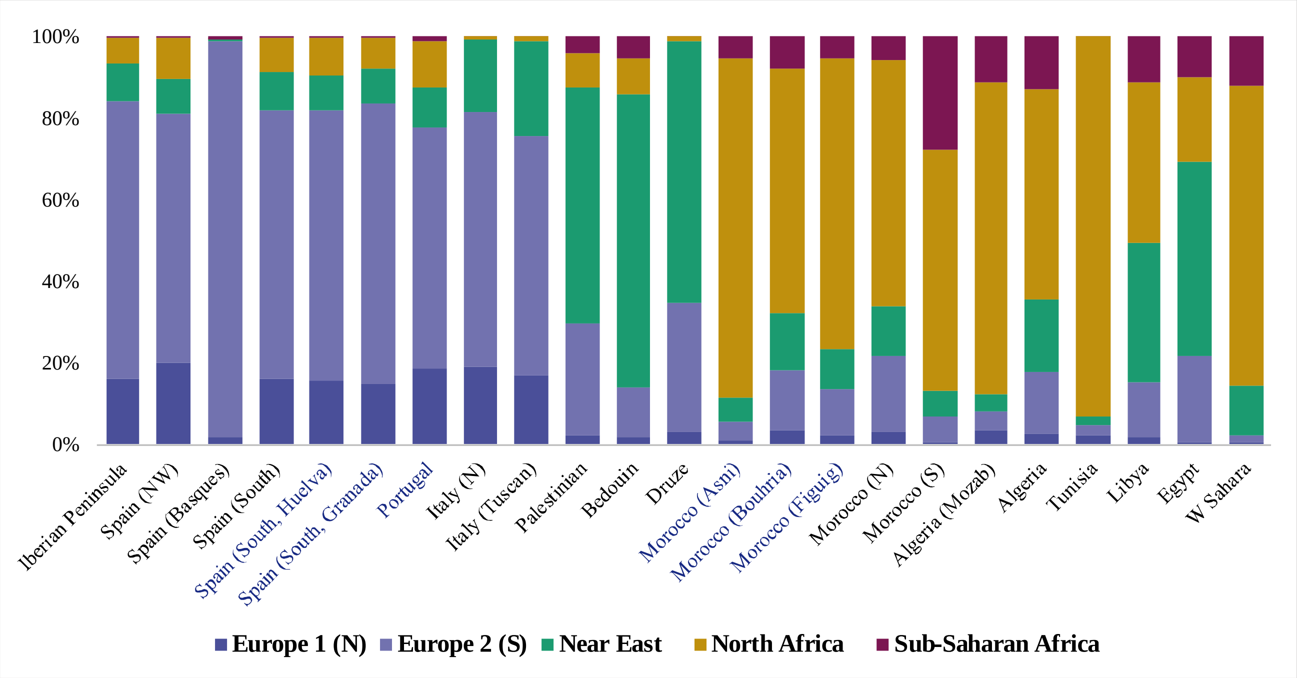

Figure S1. Mean global ancestry proportions (%) estimated through ADMIXTURE inference (K=5). See mean values in **Table S2** and details on populations selected in **Table S8**. The populations analyzed in the present study are highlighted in dark blue.

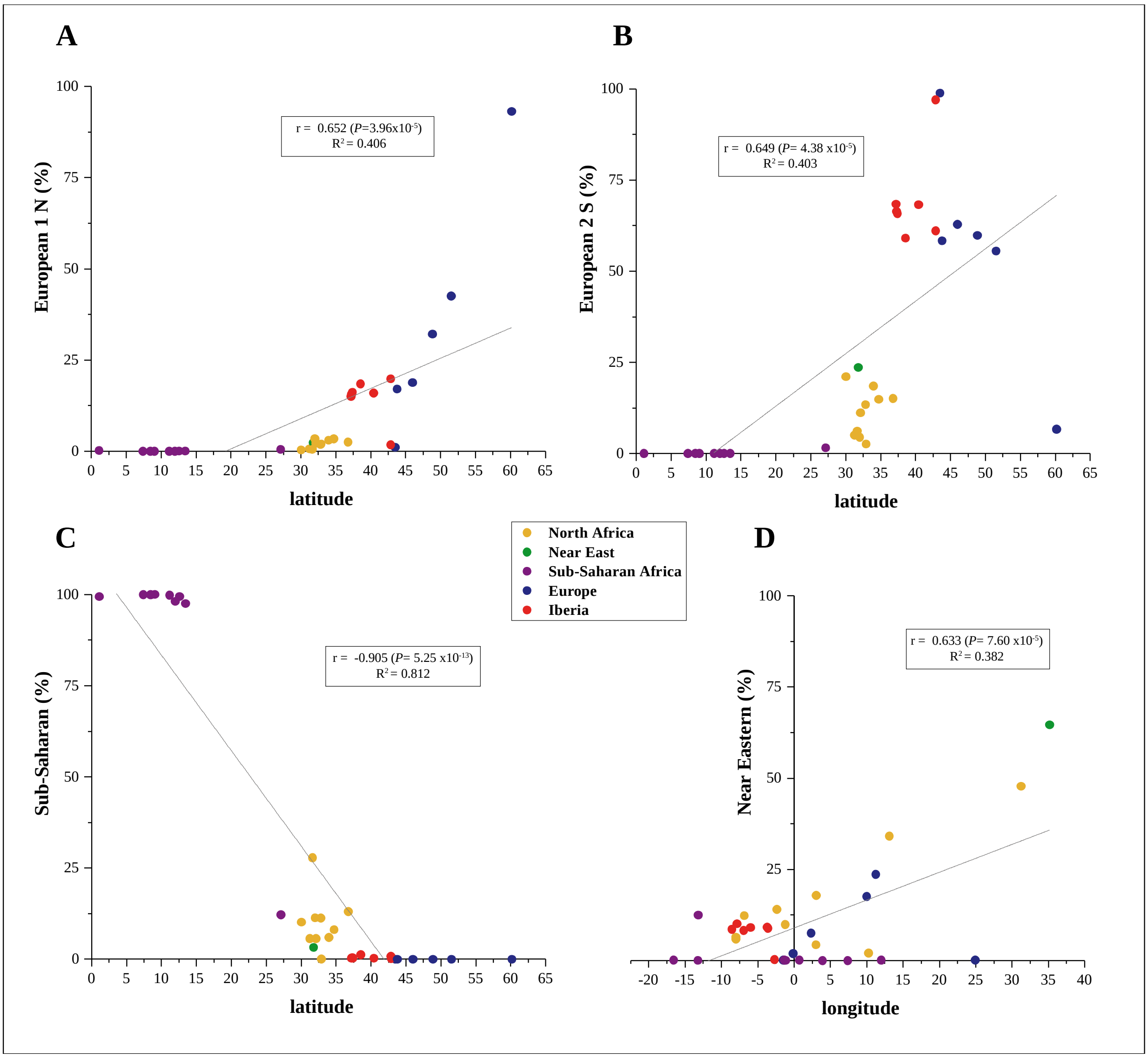

Figure S2. Correlation tests among mean values of different ancestral clusters (as inferred by ADMIXTURE K=5) and geography (Pearson's correlation coefficient). Only significant correlations were shown: **A:** Europe 1 cluster vs. latitude, **B:** Europe 2 vs. latitude, **C:** Sub-Saharan African vs. latitude and **D:** Near East vs. longitude. Color code indicates the geographic affiliation of populations (see legend).

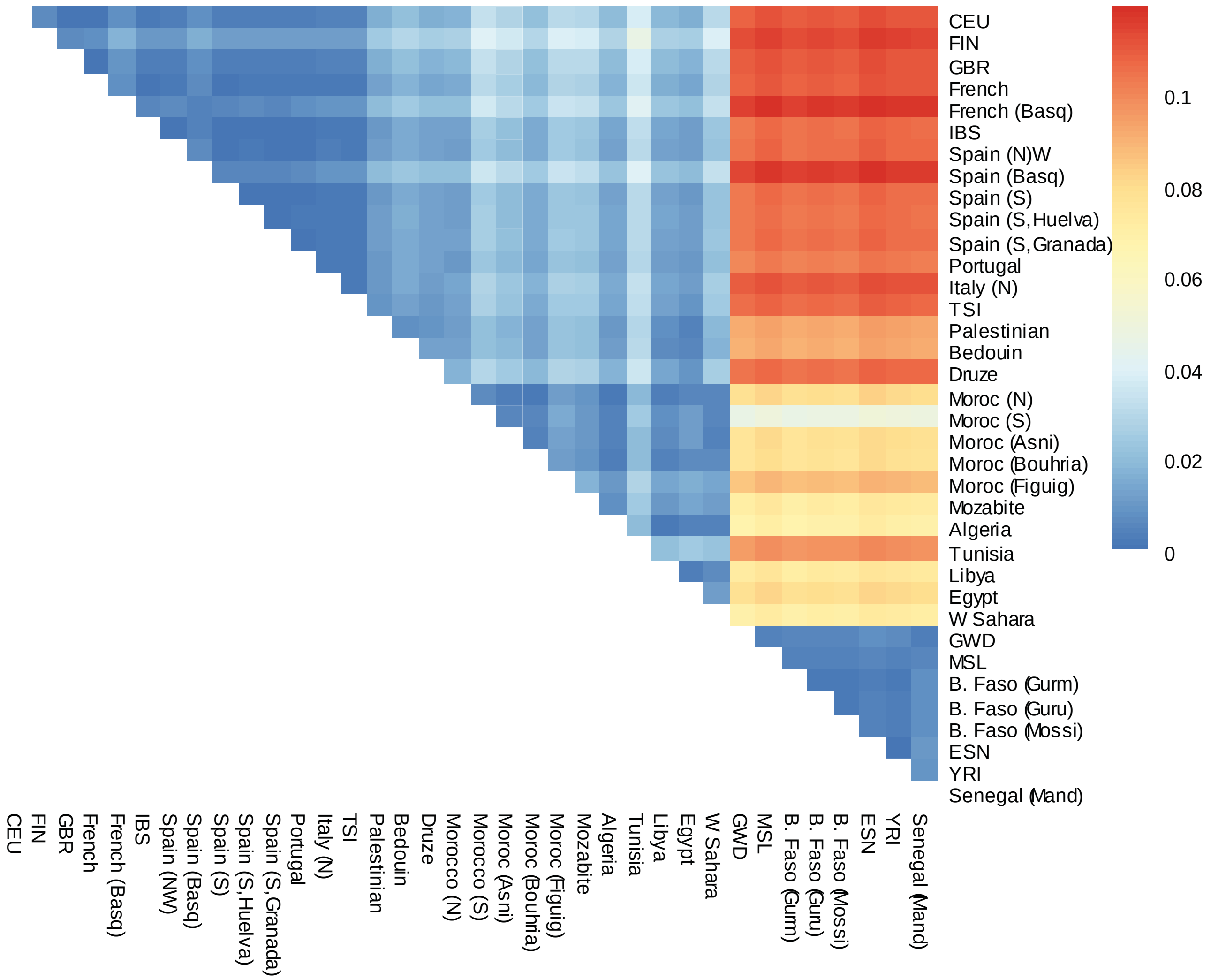

Figure S3. F_{ST} pairwise values heatmap built with R package *pheatmap*. Populations codes as in **Table S8**. Genetic distance values are coded by colors such as the legend shows.

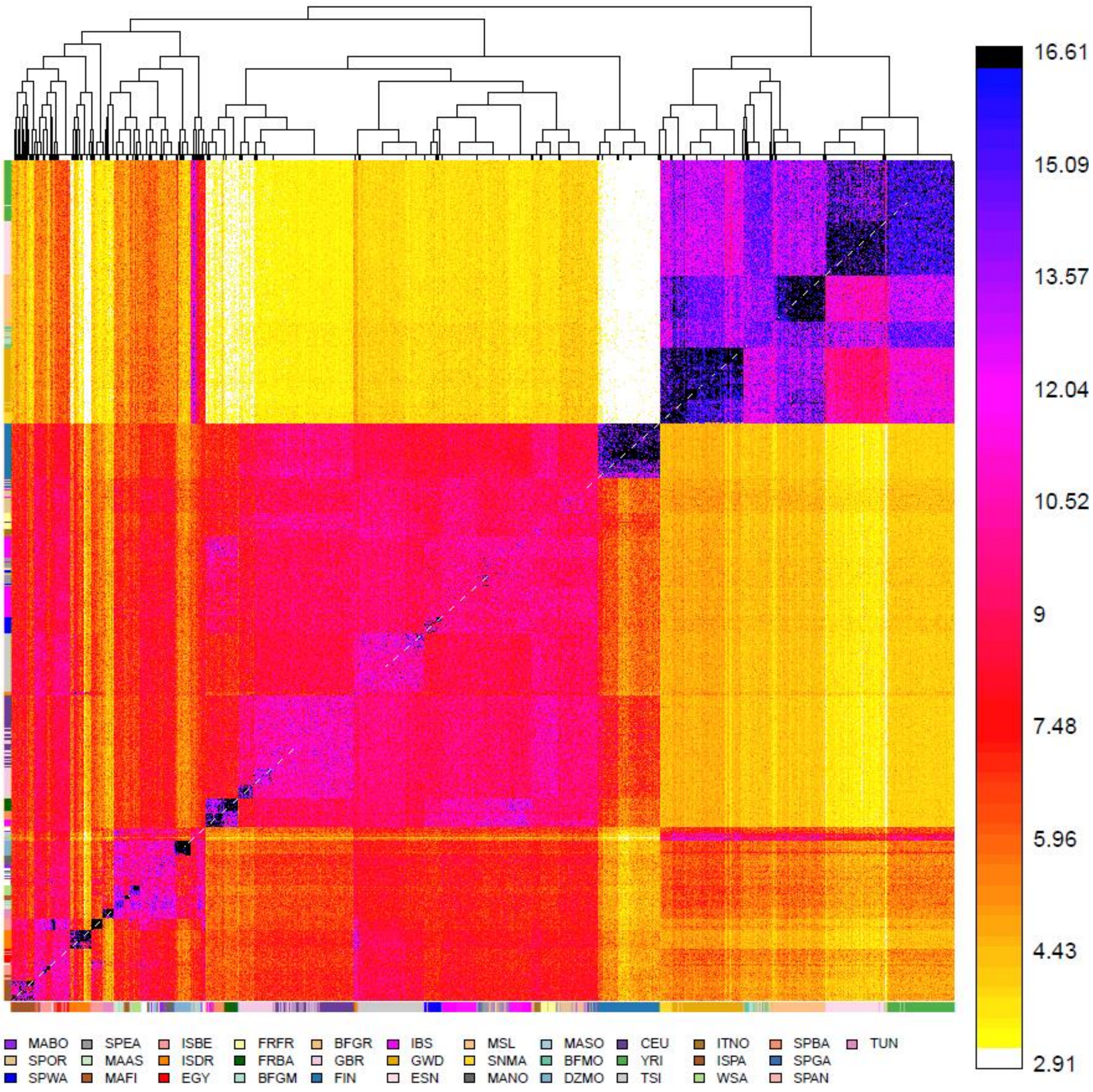

Figure S4. fineSTRUCTURE coancestry heatmap for the whole dataset (**Table S8**). Both the coancestry matrix and the tree inferred are jointly shown. Each row of the matrix represents the recipient copying vector that contains the number of chunks shared between the recipients and the donor individuals (depicted in columns).

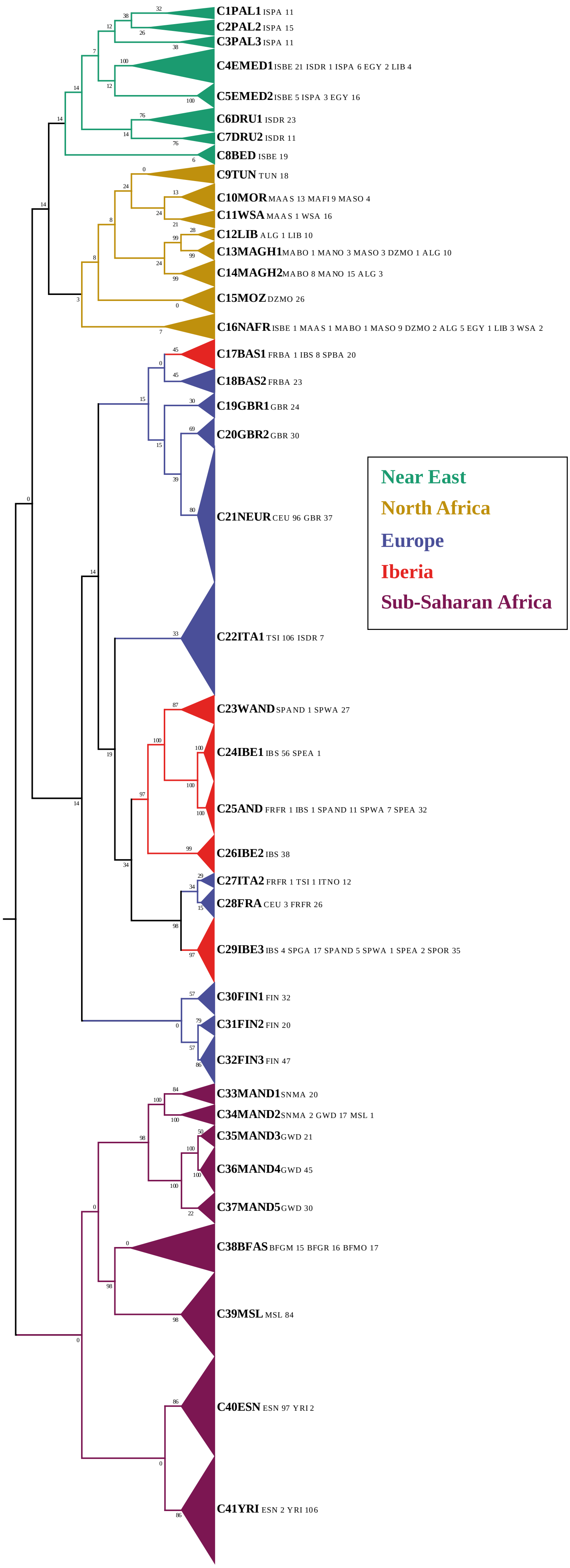

Figure S5. fineSTRUCTURE tree relating populations in different clusters. The raw tree file composed of 119 clusters was treated to reach the present tree, with 41 clusters. Each cluster is presented with its name and the number of samples belonging to the populations of the original database (see Table S8). Partition posterior probabilities are shown in branches.

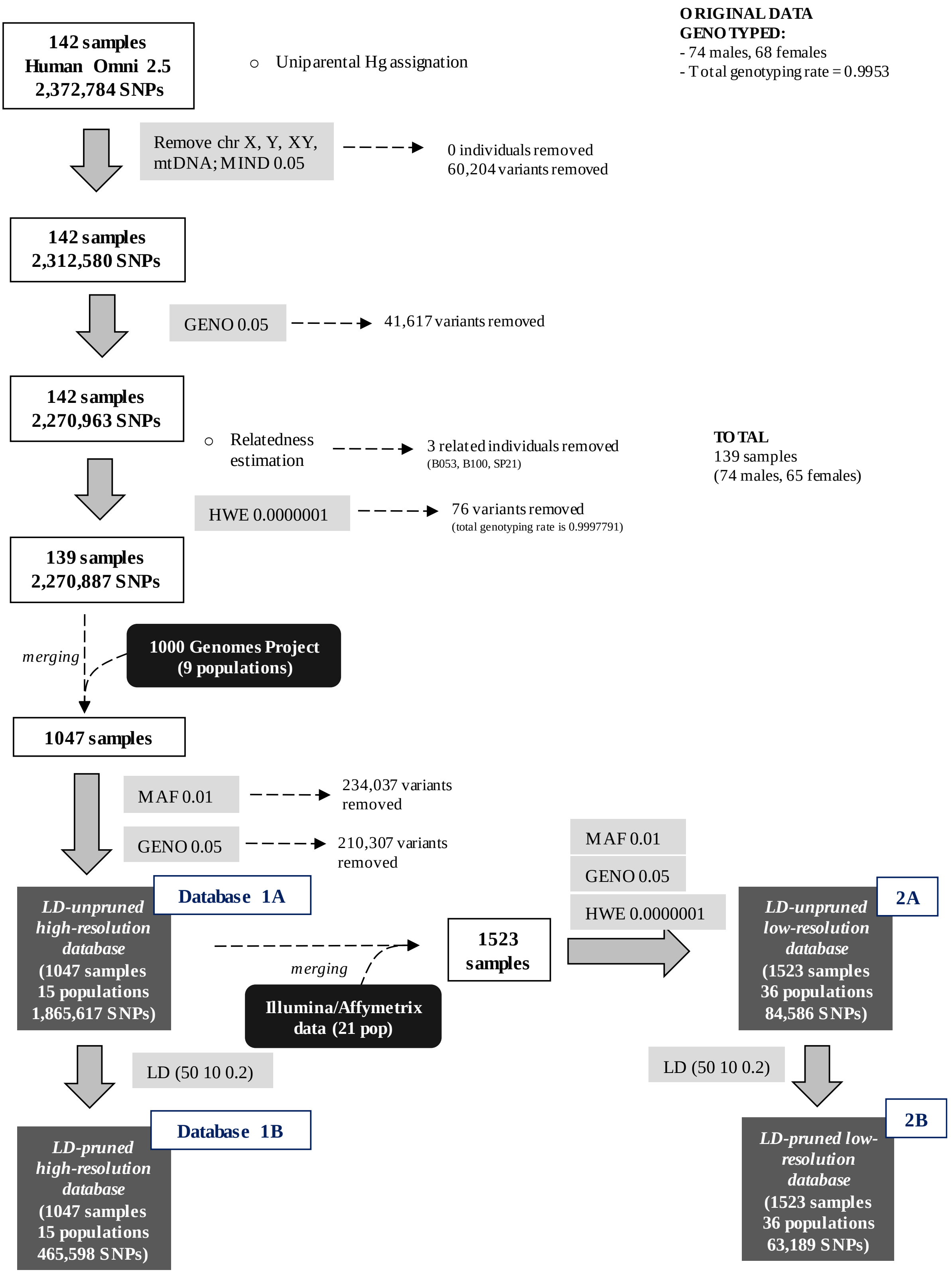

Figure S6. Schematic vision of quality control of our GW data and details on merging.

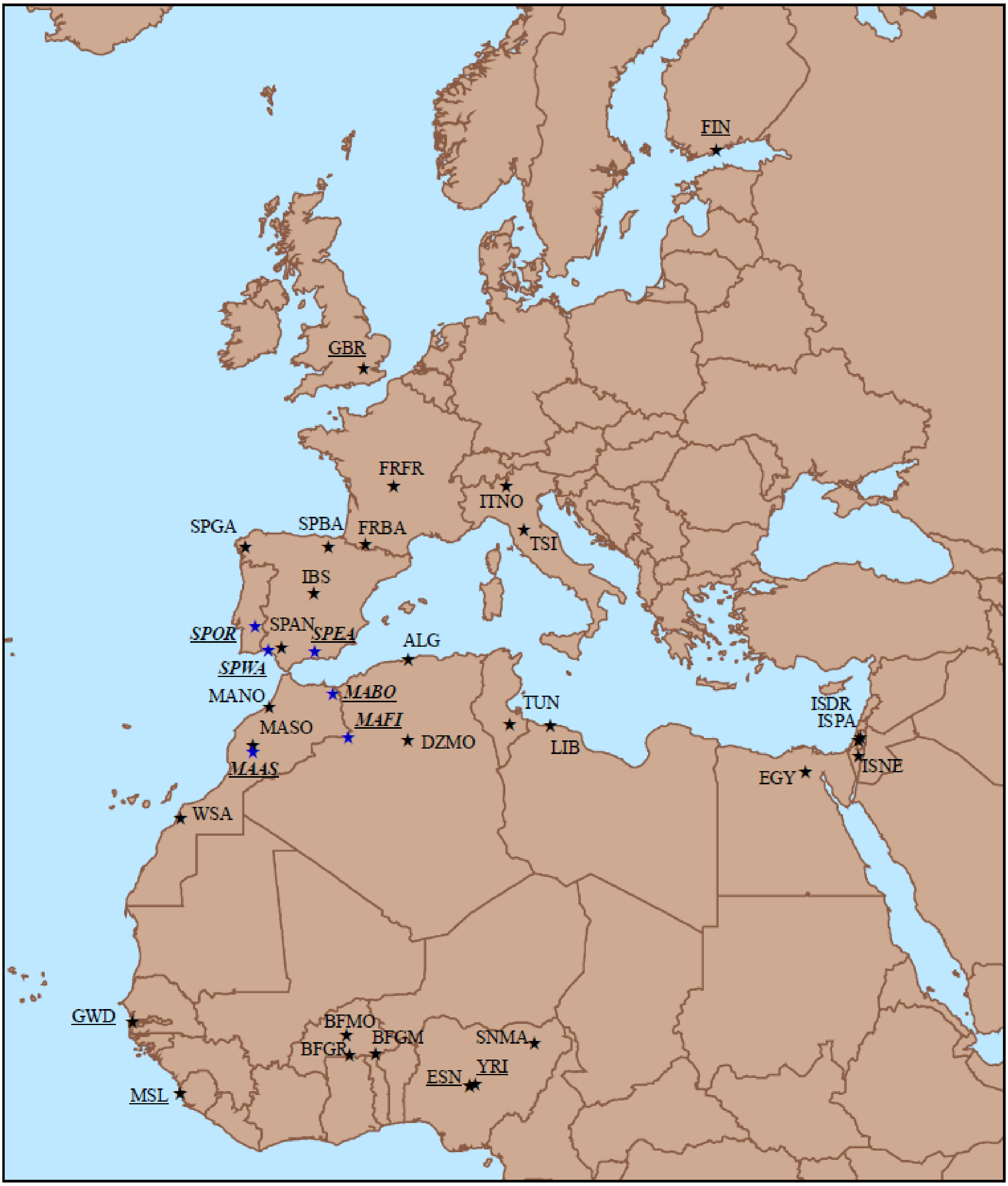

Figure S7. Map of the populations selected for the present study. CEU population was not represented as it strictly is composed of US samples of European origin. Populations ascribed to the high-density dataset (**Tables S7**) are underlined, the rest are associated to the low-density dataset (**Table S8**). The newly samples genotyped in the present study belongs to the populations highlighted in italics and bold face and marked by blue stars.

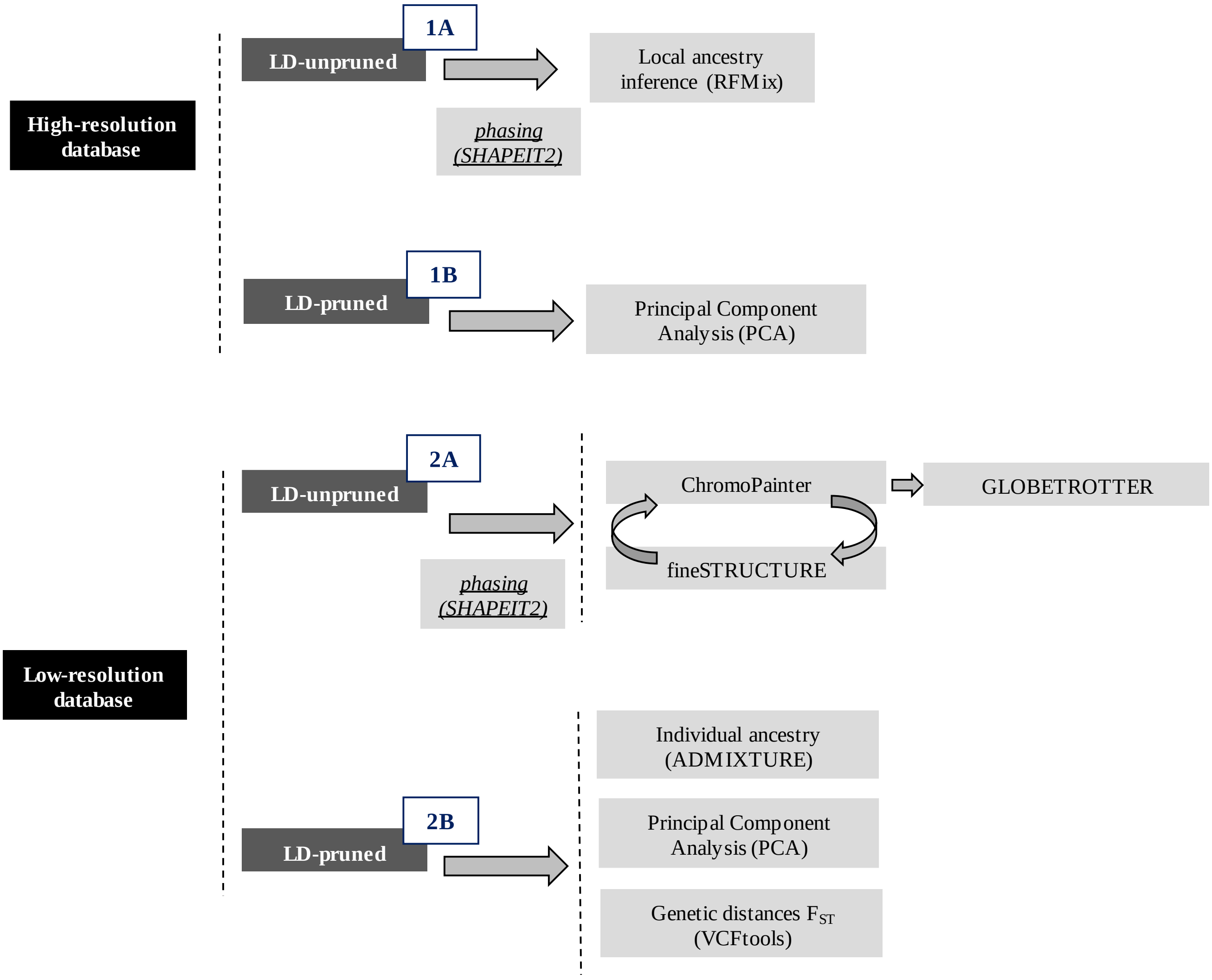

Figure S8. Bioinformatic pipeline of the analysis performed in the Databases built with different resolution.

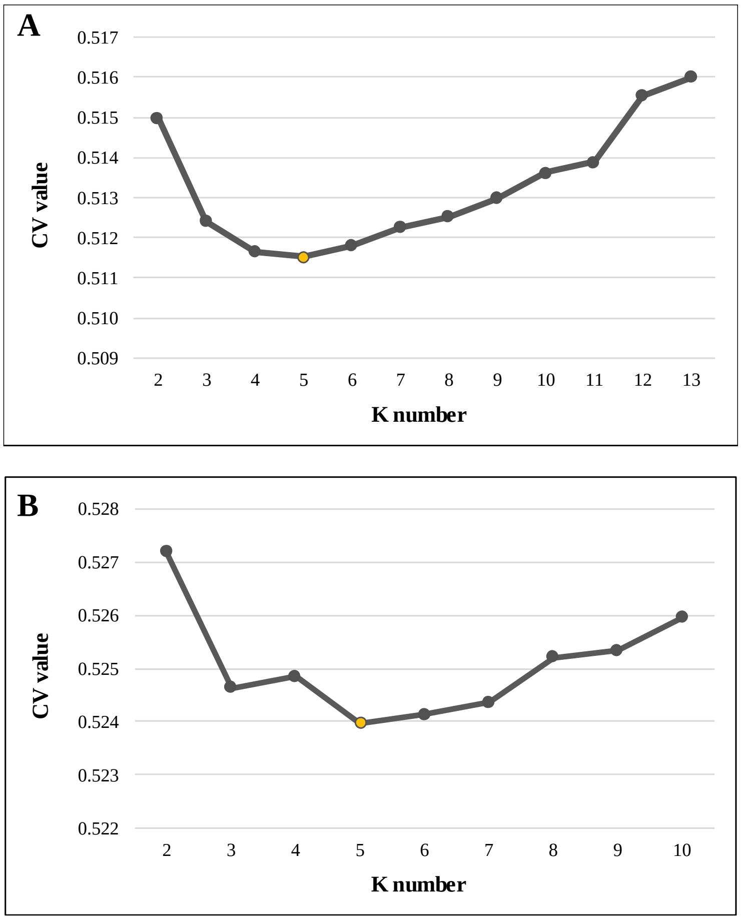

Figure S9. Cross validation test for ADMIXTURE analyses and relationship with the number of cluster inferred. **A.** Tests performed for modern dataset (**Table S8**, **Figure 2** and **Figure 3**). The lowest value (0.5115) is associated with K=5. **B.** Tests performed for both ancient and modern DNA samples (**Table S8+Table S10**, **Figure 5**). The lowest value (0.5240) is also linked to K=5.

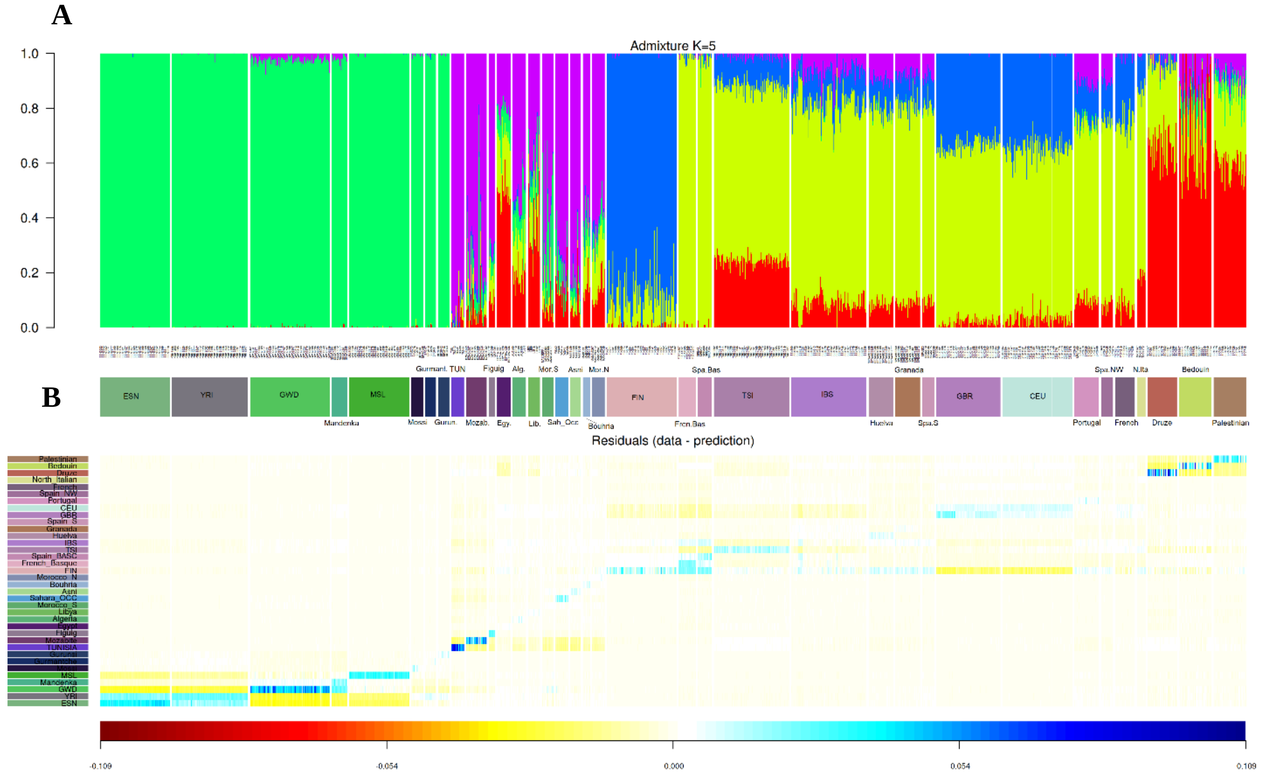

Figure S10. badMIXTURE analysis for ADMIXTURE K=5. **A.** Ancestry proportions estimated through ADMIXTURE (see populations in **Table S8**). **B.** Distribution of the residuals from the goodness of fit of the admixture model with respect to ChromoPainter chromosome painting approach.
