# Supplementary material for: Human Genomic Diversity Where the Mediterranean Joins the Atlantic
Source: Mol Biol Evol. 2019 Dec 9;37(4):1041–55. doi: 10.1093/molbev/msz288 (PMC7086172; doi:10.1093/molbev/msz288)
Supplement: msz288-Supplementary_Data [file msz288-supplementary_data.zip › msz288-Suppl_Data/MBE-19-0603_Supp Tables.pdf]

Supplementary Tables

Human genomic diversity where the Mediterranean joins the Atlantic

Candela L. Hernández, Guillermo Pita, Bruno Cavadas, Saioa López, Luis J. Sánchez-Martínez, Jean-Michel Dugoujon, Andrea Novelletto, Pedro Cuesta, Luisa Pereira & Rosario Calderón

Table S1. Mean local ancestry proportions (RFMix) 2

Table S2. Mean global ancestry proportions (ADMIXTURE K=5) 3

Table S3. Clusters defined by initial fineSTRUCTURE painting protocol 4

Table S4. Admixture dates estimated from GLOBETROTTER 5

Table S5. Samples genotyped in the present study 7

Table S6. Mitochondrial DNA haplogroups inferred 11

Table S7. High resolution database 14

Table S8. Low resolution database 15

Table S9. Scenarios tested in GLOBETROTTER 17

Table S10. Details on aDNA samples compiled 18

Table S1. Average European and African ancestral proportions (%) estimated through RFMix for parental panels of haplotypes CEU and YRI in the six population samples analyzed in the present study.

	**European ancestry (CEU panel)**		**African ancestry (YRI panel)**	
**Population**	**mean value**	**SD**	**mean value**	**SD**
Spain (SW, Andalusia)	98.79	0.53	1.21	0.53
Spain (SE, Andalusia)	99.06	0.28	0.94	0.28
Andalusia	*98.92*	*0.44*	*1.08*	*0.44*
Portugal (South)	97.91	0.71	2.09	0.71
IBERIA	*98.59*	*0.72*	*1.41*	*0.72*
Morroco (Asni Berbers)	86.26	2.98	13.74	2.98
Morroco (Bouhria Berbers)	86.31	5.22	13.69	5.22
Morroco (Figuig Berbers)	85.98	1.27	14.02	1.27
MOROCCO	*86.20*	*3.41*	*13.80*	*3.41*

SD is the standard deviation.

Table S2. Average ancestral proportions estimated through ADMIXTURE inference (K= 5) for a selected population set from Database 2B (see details and references in **Table S8**). Both northern European and sub-Saharan African populations were not considered here. Populations analyzed in the present study are highlighted in italics. SD is the standard deviation.

		**Inferred ancestral clusters**									
		**Europe 1 (N)**		**Europe 2 (S)**		**Near East**		**North Africa**		**Sub-Saharan Africa**	
**ACR**	**Population**	**%**	**SD**	**%**	**SD**	**%**	**SD**	**%**	**SD**	**%**	**SD**
**IBS**	**Iberian Peninsula**	15.96	3.76	68.27	8.00	9.13	3.29	6.38	3.72	0.27	0.54
**SPGA**	**Spain (NW, Galicia)**	19.84	2.41	61.07	3.31	8.57	2.05	10.30	1.64	0.23	0.46
**SPBA**	**Spain (Basques)**	1.77	2.42	97.00	3.76	0.27	0.73	0.19	0.50	0.76	2.02
**SPAN**	**Spain (South, Andalusia)**	16.19	2.21	65.78	3.66	9.03	2.13	8.68	2.06	0.32	0.37
SPWA	***Spain (SW, Andal. Huelva)***	*15.65*	*2.83*	*66.39*	*3.64*	*8.27*	*1.82*	*9.28*	*1.79*	*0.42*	*0.63*
SPEA	***Spain (SE, Andal. Granada)***	*15.01*	*2.91*	*68.38*	*3.68*	*8.84*	*2.01*	*7.53*	*2.23*	*0.25*	*0.43*
PTSO	***Portugal (South)***	*18.45*	*1.82*	*59.08*	*2.56*	*10.07*	*2.04*	*11.17*	*1.87*	*1.23*	*0.88*
**ITNO**	**Italy (N)**	18.82	2.48	62.82	2.43	17.59	2.21	0.77	0.79	0.00	0.00
**TSI**	**Italy (Tuscan)**	17.05	2.69	58.33	2.72	23.62	1.94	1.00	1.08	0.00	0.00
**ISPA**	**Palestinian**	2.09	2.20	27.30	3.26	58.01	4.71	8.25	3.43	4.35	2.53
**ISBE**	**Bedouin**	1.92	2.68	12.04	10.84	71.81	22.10	8.93	6.49	5.30	11.39
**ISDR**	**Druze**	3.08	2.62	31.58	4.53	64.15	5.77	1.08	1.99	0.11	0.49
MAAS	***Morocco (Asni Berbers)***	*0.67*	*1.09*	*5.05*	*2.42*	*5.91*	*6.57*	*82.74*	*8.36*	*5.64*	*3.70*
MABO	***Morocco (Bouhria Berbers)***	*3.41*	*2.50*	*14.87*	*4.90*	*14.03*	*4.96*	*59.58*	*5.42*	*8.11*	*5.29*
MAFI	***Morocco (Figuig Berbers)***	*2.17*	*2.59*	*11.19*	*2.28*	*9.86*	*2.64*	*71.16*	*3.76*	*5.62*	*1.59*
**MANO**	**Morocco (North)**	3.02	2.55	18.48	4.82	12.30	5.06	60.26	5.35	5.94	2.46
**MASO**	**Morocco (South)**	0.53	0.86	6.16	3.37	6.43	4.20	59.09	19.86	27.78	20.87
**DZMO**	**Algeria (Mozabites)**	3.46	1.98	4.45	5.34	4.31	4.91	76.45	17.80	11.34	15.18
**ALG**	**Algeria**	2.49	2.20	15.10	4.46	17.84	4.59	51.51	6.64	13.06	9.28
**TUN**	**Tunisia (Chenini)**	2.01	1.39	2.61	3.58	2.04	3.03	93.31	6.22	0.04	0.16
**LIB**	**Libya**	1.88	2.41	13.43	3.52	34.11	10.35	39.34	8.45	11.25	7.09
**EGY**	**Egypt**	0.38	0.91	21.08	3.07	47.81	4.62	20.58	2.70	10.15	4.05
**WSA**	**W Sahara**	0.49	1.01	1.57	2.22	12.49	5.72	73.26	13.36	12.20	12.87

Table S3. Clusters defined by the initial painting protocol of fineSTRUCTURE (N=1523) performing an analysis all vs. all (all the individuals acted both as recipients and donors). The population composition of each cluster is shown in the second column. Codes as in **Table S8**. Clusters are classified in colors depending on their geographic affiliation [green: Near East (N=148), yellow: North Africa (N=167), blue: Europe (N=733), purple: sub-Saharan Africa (N=475)].

Cluster	Name	N
C1PAL1	**PALESTINA 1** ISPA (11)	11
C2PAL2	**PALESTINA 2** ISPA (15)	15
C3PAL3	**PALESTINA 3** ISPA (11)	11
C4EMED1	**EAST MEDITERRANEAN 1** ISBE (21) ISDR (1) ISPA (6) EGY (2) LIB (4)	34
C5EMED2	**EAST MEDITERRANEAN 2** ISBE (5) ISPA (3) EGY (16)	24
C6DRU1	**DRUZE 1** ISDR (23)	23
C7DRU2	**DRUZE 2** ISDR (11)	11
C8BED	**BEDOUIN** ISBE (19)	19
C9TUN	**TUNISIA** TUN (18)	18
C10MOR	**MOROCCO** MAAS (13) MAFI (9) MASO (4)	26
C11WSA	**W SAHARA** MAAS (1) WSA (16)	17
C12LIB	**LIBYA** ALG (1) LIB (10)	11
C13MAGH1	**MAGHREB 1** MABO (1) MANO (3) MASO (3) DZMO (1) ALG (10)	18
C14MAGH2	**MAGHREB 2** MABO (8) MANO (15) ALG (3)	26
C15MOZ	**MOZABITES** DZMO (26)	26
C16NAFR	**NORTH AFRICA** ISBE (1) MAAS (1) MABO (1) MASO (9) DZMO (2) ALG (5) EGY (1) LIB (3) WSA (2)	25
C17BAS1	**BASQUES 1** FRBA (1) IBS (8) SPBA (20)	29
C18BAS2	**BASQUES 2** FRBA (23)	23
C19GBR1	**G BRITAIN 1** GRB (24)	24
C20GBR2	**G BRITAIN 2** GBR (30)	30
C21NEUR	**NC EUROPE** CEU (96) GBR (37)	133
C22ITA1	**ITALY 1** TSI (106) ISDR (7)	113
C23WAND	**W ANDALUSIA** SPAN (1) SPWA (27)	28
C24IBE1	**IBERIA 1** IBS (56) SPEA (1)	57
C25AND	**ANDALUSIA** FRFR (1) IBS (1) SPAN (11) SPWA (7) SPEA (32)	52
C26IBE2	**IBERIA 2** IBS (38)	38
C27ITA2	**ITALY 2** FRFR (1) TSI (1) ITNO (12)	14
C28FRA	**FRANCE** CEU (3) FRFR (26)	29
C29IBE3	**IBERIA 3** IBS (4) SPGA (17) SPAN (5) SPWA (1) SPEA (2) SPOR (35)	64
C30FIN1	**FINLAND 1** FIN (32)	32
C31FIN2	**FINLAND 2** FIN (20)	20
C32FIN3	**FINLAND 3** FIN (47)	47
C33MAND1	**MANDINKA 1** SNMA (20)	20
C34MAND2	**MANDINKA 2** SNMA (2) GWD (17) MSL (1)	20
C35MAND3	**MANDINKA 3** GWD (21)	21
C36MAND4	**MANDINKA 4** GWD (45)	45
C37MAND5	**MANDINKA 5** GWD (30)	30
C38BFAS	**B FASO** BFGM (15) BFGR (16) BFMO (17)	48
C39MSL	**S LEONE** MSL (84)	84
C40ESN	**NIGERIA 1** ESN (97) YRI (2)	99
C41YRI	**NIGERIA 2** ESN (2) YRI (106)	108

Table S4. Admixture dates estimated from GLOBETROTTER (calculated as 2000 - 25 x generation time) for the different migratory scenarios tested (see **Table S9**). For the Iberian recipients, donor clusters were as follows: test A (C4EMED1, C14MAGH2, C21NEUR, C41YRI), test B (C4EMED1, C10MOR, C21NEUR, C41YRI). For North African recipients: test A (C4EMED1, C22ITA1, C25AND, C41YRI), test C (C4EMED1, C22ITA1, C23WAND, C41YRI), test D (C4EMED1, C22ITA1, C24IBE1, C41YRI) and test E (C4EMED1, C22ITA1, C29IBE3, C41YRI). The scenarios selected for **Figure 4** (main manuscript) were highlighted in color. **A.** Clusters that suffered one-date admixture events according to GLOBETROTTER. **B.** Clusters where two-date admixture events were inferred.

A. One-date admixture events

	Target	Date (95% CI)	Prob. source 1 (clusters > 5%)	Best source 1	Prob. source 2 (clusters > 5%)	Best source 2
Iberia	**C23WAND**	1088 (1114-1062)	0.09 (C14MAGH2:0.05)	C14MAGH2	0.91 (C14MAGH2:0.05; C4EMED1:0.08; C21NEUR:0.77)	C21NEUR
	**C23WANDb**	1203 (1228-1179)	0.11 (C10MOR:0.06)	C10MOR	0.89 (C4EMED1:0.08; C21NEUR:0.78)	C21NEUR
	**C24IBE1**	1101 (1126-1075)	0.24 (C4EMED1:0.05; C14MAGH2:0.19)	C14MAGH2	0.76 (C4EMED1:0.05; C21NEUR:0.71)	C21NEUR
	**C24IBE1b**	1081 (1106-1057)	0.19 (C4EMED1:0.09; C10MOR:0.10)	C21NEUR	0.81 (C4EMED1:0.06; C21NEUR:0.73)	C21NEUR
	**C25AND**	1143 (1162-1124)	0.25 (C4EMED1:0.06; C14MAGH2:0.19)	C14MAGH2	0.75 (C21NEUR:0.71)	C21NEUR
	**C25ANDb**	1016 (1039-993)	0.11 (C10MOR:0.05)	C10MOR	0.89 (C4EMED1:0.08; C21NEUR:0.78)	C21NEUR
	**C26IBE2**	1110 (1136-1083)	0.23 (C4EMED1:0.07; C14MAGH2:0.15)	C14MAGH2	0.77 (C21NEUR:0.72)	C21NEUR
	**C26IBE2b**	1071 (1096-1046)	0.20 (C10MOR:0.10; C4EMED1:0.10)	C21NEUR	0.80 (C21NEUR:0.75)	C21NEUR
North Africa	**C10MOR**	1294 (1314-1273)	0.28 (C4EMED1:0.05; C25AND:0.08; C41YRI:0.15)	C41YRI	0.72 (C4EMED1:0.24; C25AND:0.47)	C22ITA1
	**C10MORc**	1307 (1328-1287)	0.32 (C23WAND:0.07; C4EMED1:0.09; C41YRI:0.16)	C41YRI	0.68 (C23WAND:0.21; C4EMED1:0.23; C22ITA1:0.24)	C22ITA1
	**C10MORd**	1267 (1292-1242)	0.26 (C24IBE1:0.08; C41YRI:0.14)	C41YRI	0.74 (C4EMED1:0.21; C24IBE1:0.52)	C22ITA1
	**C10MORe**	1314 (1333-1295)	0.18 (C29IBE3:0.05; C41YRI:0.11)	C41YRI	0.82 (C4EMED1:0.22; C29IBE3:0.57)	C29IBE3
	**C11WSA**	1548 (1558-1539)	0.18 (C41YRI:0.13)	C41YRI	0.82 (C4EMED1:0.37; C25AND:0.42)	C22ITA1
	**C11WSAc**	1489 (1501-1477)	0.28 (C23WAND:0.06; C41YRI:0.17)	C41YRI	0.72 (C22ITA1:0.17; C23WAND:0.18; C4EMED1:0.37)	C22ITA1
	**C11WSAd**	1517 (1526-1509)	0.15 (C41YRI:0.12)	C41YRI	0.85 (C41YRI:0.05; C4EMED1:0.34; C24IBE1:0.46)	C22ITA1
	**C11WSAe**	1556 (1565-1547)	0.22 (C29IBE3:0.06; C41YRI:0.14)	C41YRI	0.78 (C4EMED1:0.31; C29IBE3:0.45)	C22ITA1
	**C13MAGH1**	1440 (1449-1431)	0.19 (C41YRI:0.16)	C41YRI	0.81 (C22ITA1:0.11; C4EMED1:0.31; C25AND:0.38)	C22ITA1
	**C13MAGH1c**	1451 (1461-1441)	0.18 (C41YRI:0.15)	C41YRI	0.82 (C23WAND:0.20; C22ITA1:0.28; C4EMED1:0.33)	C22ITA1
	**C13MAGH1d**	1426 (1435-1416)	0.19 (C41YRI:0.15)	C41YRI	0.81 (C4EMED1:0.28; C24IBE1:0.53)	C22ITA1
	**C13MAGH1e**	1461 (1471-1451)	0.18 (C41YRI:0.15)	C41YRI	0.82 (C4EMED1:0.29; C29IBE3:0.53)	C22ITA1
	**C14MAGH2**	1276 (1288-1265)	0.20 (C41YRI:0.13)	C41YRI	0.80 (C22ITA1:0.12; C4EMED1:0.20; C25AND:0.47)	C22ITA1
	**C14MAGH2c**	1164 (1177-1151)	0.20 (C4EMED1:0.05; C41YRI:0.14)	C41YRI	0.80 (C4EMED1:0.23; C23WAND:0.25; C22ITA1:0.32)	C22ITA1
	**C14MAGH2d**	1268 (1280-1256)	0.16 (C41YRI:0.11)	C41YRI	0.84 (C4EMED1:0.19; C24IBE1:0.63)	C29IBE3
	**C14MAGH2e**	1219 (1231-1207)	0.14 (C41YRI:0.11)	C41YRI	0.86 (C4EMED1:0.20; C29IBE3:0.65)	C29IBE3

Table S4. Cont.

B. Multiple-date admixture events

		1st Event					2nd Event				
	**Target**	**Date (95% CI)**	**Prob. source 1 (clusters >5%)**	**Best source 1**	**Prob. source 2 (clusters >5%)**	**Best source 2**	**Date (95% CI)**	**Prob. source 1 (clusters >5%)**	**Best source 1**	**Prob. source 2 (clusters >5%)**	**Best source 2**
**Iberia**	**C29IBE3**	1750 (1766-1733)	0.18 (C21NEUR:0.13)	C14MAGH2	0.82 (C14MAGH2:0.09; C4EMED1:0.16; C21NEUR:0.57)	C21NEUR	890 (926-853)	0.21 (C4EMED1:0.06; C14MAGH2:0.12)	C14MAGH2	0.79 (C21NEUR:0.74)	C21NEUR
	**C29IBE3b**	1766 (1776-1756)	0.07	C4EMED1	0.93 (C10MOR:0.08; C4EMED1:0.15; C21NEUR:0.70)	C21NEUR	656 (681-630)	0.17 (C10MOR:0.09)	C10MOR	0.83 (C4EMED1:0.05; C21NEUR:0.77)	C21NEUR
**North Africa**	**C16NAFR**	1891 (1901-1882)	0.40 (C22ITA1:0.08; C41YRI:0.29)	C41YRI	0.60 (C41YRI:0.10; C4EMED1:0.21; C25AND:0.29)	C4EMED1	1270 (1295-1244)	0.45 (C4EMED1:0.15; C25AND:0.29)	C22ITA1	0.55 (C22ITA1:0.06; C4EMED1:0.10; C41YRI:0.39)	C41YRI
	**C16NAFRc**	1877 (1887-1866)	0.50 (C41YRI:0.06; C23WAND:0.12; C22ITA1:0.13; C4EMED1:0.19)	C4EMED1	0.50 (C22ITA1:0.06; C4EMED1:0.07; C41YRI:0.34)	C41YRI	1248 (1272-1224)	0.44 (C23WAND:0.08; C4EMED1:0.17; C22ITA1:0.19)	C22ITA1	0.56 (C23WAND:0.07; C4EMED1:0.10; C41YRI:0.39)	C41YRI
	**C16NAFRd**	1864 (1874-1854)	0.42 (C24IBE1:0.06; C4EMED1:0.06; C41YRI:0.30)	C41YRI	0.58 (C41YRI:0.09; C4EMED1:0.16; C24IBE1:0.33)	C4EMED1	1212 (1243-1182)	0.32 (C41YRI:0.28)	C41YRI	0.68 (C41YRI:0.11; C4EMED1:0.18; C24IBE1:0.39)	C4EMED1
	**C16NAFRe**	1861 (1869-1853)	0.31 (C41YRI:0.25)	C41YRI	0.69 (C41YRI:0.13; C4EMED1:0.18; C29IBE3:0.38)	C4EMED1	1199 (1225-1172)	0.45 (C4EMED1:0.07; C41YRI:0.34)	C41YRI	0.55 (C4EMED1:0.15; C29IBE3:0.35)	C29IBE3

Table S5. Details on the samples genotyped in the present study.

Sample ID	Code	ACR	Country	Region	Population	Sex
299532	SPWA299532	SPWA	Spain	Southwestern Europe	Western Andalusia (Huelva)	Female
299590	SPWA299590	SPWA	Spain	Southwestern Europe	Western Andalusia (Huelva)	Female
299593	SPWA299593	SPWA	Spain	Southwestern Europe	Western Andalusia (Huelva)	Female
299601	SPWA299601	SPWA	Spain	Southwestern Europe	Western Andalusia (Huelva)	Male
299605	SPWA299605	SPWA	Spain	Southwestern Europe	Western Andalusia (Huelva)	Female
334176	SPWA334176	SPWA	Spain	Southwestern Europe	Western Andalusia (Huelva)	Female
334183	SPWA334183	SPWA	Spain	Southwestern Europe	Western Andalusia (Huelva)	Female
334186	SPWA334186	SPWA	Spain	Southwestern Europe	Western Andalusia (Huelva)	Female
334200	SPWA334200	SPWA	Spain	Southwestern Europe	Western Andalusia (Huelva)	Male
334206	SPWA334206	SPWA	Spain	Southwestern Europe	Western Andalusia (Huelva)	Female
334210	SPWA334210	SPWA	Spain	Southwestern Europe	Western Andalusia (Huelva)	Female
334211	SPWA334211	SPWA	Spain	Southwestern Europe	Western Andalusia (Huelva)	Male
334230	SPWA334230	SPWA	Spain	Southwestern Europe	Western Andalusia (Huelva)	Female
334240	SPWA334240	SPWA	Spain	Southwestern Europe	Western Andalusia (Huelva)	Male
334280	SPWA334280	SPWA	Spain	Southwestern Europe	Western Andalusia (Huelva)	Male
334282	SPWA334282	SPWA	Spain	Southwestern Europe	Western Andalusia (Huelva)	Female
335011	SPWA335011	SPWA	Spain	Southwestern Europe	Western Andalusia (Huelva)	Female
335044	SPWA335044	SPWA	Spain	Southwestern Europe	Western Andalusia (Huelva)	Male
335066	SPWA335066	SPWA	Spain	Southwestern Europe	Western Andalusia (Huelva)	Male
335069	SPWA335069	SPWA	Spain	Southwestern Europe	Western Andalusia (Huelva)	Male
335077	SPWA335077	SPWA	Spain	Southwestern Europe	Western Andalusia (Huelva)	Female
335081	SPWA335081	SPWA	Spain	Southwestern Europe	Western Andalusia (Huelva)	Male
335088	SPWA335088	SPWA	Spain	Southwestern Europe	Western Andalusia (Huelva)	Male
335097	SPWA335097	SPWA	Spain	Southwestern Europe	Western Andalusia (Huelva)	Female
335114	SPWA335114	SPWA	Spain	Southwestern Europe	Western Andalusia (Huelva)	Female
601206	SPWA601206	SPWA	Spain	Southwestern Europe	Western Andalusia (Huelva)	Female
601215	SPWA601215	SPWA	Spain	Southwestern Europe	Western Andalusia (Huelva)	Male
601238	SPWA601238	SPWA	Spain	Southwestern Europe	Western Andalusia (Huelva)	Male
602790	SPWA602790	SPWA	Spain	Southwestern Europe	Western Andalusia (Huelva)	Male
602797	SPWA602797	SPWA	Spain	Southwestern Europe	Western Andalusia (Huelva)	Male
602821	SPWA602821	SPWA	Spain	Southwestern Europe	Western Andalusia (Huelva)	Female
602822	SPWA602822	SPWA	Spain	Southwestern Europe	Western Andalusia (Huelva)	Male
602825	SPWA602825	SPWA	Spain	Southwestern Europe	Western Andalusia (Huelva)	Male
602829	SPWA602829	SPWA	Spain	Southwestern Europe	Western Andalusia (Huelva)	Male
602831	SPWA602831	SPWA	Spain	Southwestern Europe	Western Andalusia (Huelva)	Female
930298	SPEA930298	SPEA	Spain	Southwestern Europe	Eastern Andalusia (Granada)	Female
930306	SPEA930306	SPEA	Spain	Southwestern Europe	Eastern Andalusia (Granada)	Male
930359	SPEA930359	SPEA	Spain	Southwestern Europe	Eastern Andalusia (Granada)	Male
930374	SPEA930374	SPEA	Spain	Southwestern Europe	Eastern Andalusia (Granada)	Female
930382	SPEA930382	SPEA	Spain	Southwestern Europe	Eastern Andalusia (Granada)	Female
930400	SPEA930400	SPEA	Spain	Southwestern Europe	Eastern Andalusia (Granada)	Female

Sample ID	Code	ACR	Country	Region	Population	Sex
930401	SPEA930401	SPEA	Spain	Southwestern Europe	Eastern Andalusia (Granada)	Male
932167	SPEA932167	SPEA	Spain	Southwestern Europe	Eastern Andalusia (Granada)	Female
932176	SPEA932176	SPEA	Spain	Southwestern Europe	Eastern Andalusia (Granada)	Male
932212	SPEA932212	SPEA	Spain	Southwestern Europe	Eastern Andalusia (Granada)	Male
932232	SPEA932232	SPEA	Spain	Southwestern Europe	Eastern Andalusia (Granada)	Male
932273	SPEA932273	SPEA	Spain	Southwestern Europe	Eastern Andalusia (Granada)	Male
932362	SPEA932362	SPEA	Spain	Southwestern Europe	Eastern Andalusia (Granada)	Male
932396	SPEA932396	SPEA	Spain	Southwestern Europe	Eastern Andalusia (Granada)	Female
932404	SPEA932404	SPEA	Spain	Southwestern Europe	Eastern Andalusia (Granada)	Female
932406	SPEA932406	SPEA	Spain	Southwestern Europe	Eastern Andalusia (Granada)	Female
932408	SPEA932408	SPEA	Spain	Southwestern Europe	Eastern Andalusia (Granada)	Male
932431	SPEA932431	SPEA	Spain	Southwestern Europe	Eastern Andalusia (Granada)	Female
975465	SPEA975465	SPEA	Spain	Southwestern Europe	Eastern Andalusia (Granada)	Male
975478	SPEA975478	SPEA	Spain	Southwestern Europe	Eastern Andalusia (Granada)	Female
975512	SPEA975512	SPEA	Spain	Southwestern Europe	Eastern Andalusia (Granada)	Female
975546	SPEA975546	SPEA	Spain	Southwestern Europe	Eastern Andalusia (Granada)	Male
975563	SPEA975563	SPEA	Spain	Southwestern Europe	Eastern Andalusia (Granada)	Male
975568	SPEA975568	SPEA	Spain	Southwestern Europe	Eastern Andalusia (Granada)	Female
975581	SPEA975581	SPEA	Spain	Southwestern Europe	Eastern Andalusia (Granada)	Male
975584	SPEA975584	SPEA	Spain	Southwestern Europe	Eastern Andalusia (Granada)	Male
975606	SPEA975606	SPEA	Spain	Southwestern Europe	Eastern Andalusia (Granada)	Male
988330	SPEA988330	SPEA	Spain	Southwestern Europe	Eastern Andalusia (Granada)	Male
988339	SPEA988339	SPEA	Spain	Southwestern Europe	Eastern Andalusia (Granada)	Female
988360	SPEA988360	SPEA	Spain	Southwestern Europe	Eastern Andalusia (Granada)	Female
988368	SPEA988368	SPEA	Spain	Southwestern Europe	Eastern Andalusia (Granada)	Male
988385	SPEA988385	SPEA	Spain	Southwestern Europe	Eastern Andalusia (Granada)	Female
988393	SPEA988393	SPEA	Spain	Southwestern Europe	Eastern Andalusia (Granada)	Female
988396	SPEA988396	SPEA	Spain	Southwestern Europe	Eastern Andalusia (Granada)	Male
988408	SPEA988408	SPEA	Spain	Southwestern Europe	Eastern Andalusia (Granada)	Male
SP01	SPOR01	SPOR	Portugal	Southwestern Europe	Southern Portugal	Female
SP02	SPOR02	SPOR	Portugal	Southwestern Europe	Southern Portugal	Female
SP04	SPOR04	SPOR	Portugal	Southwestern Europe	Southern Portugal	Female
SP05	SPOR05	SPOR	Portugal	Southwestern Europe	Southern Portugal	Male
SP06	SPOR06	SPOR	Portugal	Southwestern Europe	Southern Portugal	Male
SP07	SPOR07	SPOR	Portugal	Southwestern Europe	Southern Portugal	Male
SP08	SPOR08	SPOR	Portugal	Southwestern Europe	Southern Portugal	Male
SP09	SPOR09	SPOR	Portugal	Southwestern Europe	Southern Portugal	Female
SP11	SPOR11	SPOR	Portugal	Southwestern Europe	Southern Portugal	Female
SP12	SPOR12	SPOR	Portugal	Southwestern Europe	Southern Portugal	Female
SP14	SPOR14	SPOR	Portugal	Southwestern Europe	Southern Portugal	Male
SP15	SPOR15	SPOR	Portugal	Southwestern Europe	Southern Portugal	Female
SP150	SPOR150	SPOR	Portugal	Southwestern Europe	Southern Portugal	Female
SP16	SPOR16	SPOR	Portugal	Southwestern Europe	Southern Portugal	Female
SP18	SPOR18	SPOR	Portugal	Southwestern Europe	Southern Portugal	Male

Sample ID	Code	ACR	Country	Region	Population	Sex
SP19	SPOR19	SPOR	Portugal	Southwestern Europe	Southern Portugal	Male
SP20	SPOR20	SPOR	Portugal	Southwestern Europe	Southern Portugal	Male
SP21	SPOR21	SPOR	Portugal	Southwestern Europe	Southern Portugal	Female
SP23	SPOR23	SPOR	Portugal	Southwestern Europe	Southern Portugal	Male
SP25	SPOR25	SPOR	Portugal	Southwestern Europe	Southern Portugal	Female
SP26	SPOR26	SPOR	Portugal	Southwestern Europe	Southern Portugal	Male
SP27	SPOR27	SPOR	Portugal	Southwestern Europe	Southern Portugal	Male
SP28	SPOR28	SPOR	Portugal	Southwestern Europe	Southern Portugal	Male
SP29	SPOR29	SPOR	Portugal	Southwestern Europe	Southern Portugal	Male
SP31	SPOR31	SPOR	Portugal	Southwestern Europe	Southern Portugal	Male
SP33	SPOR33	SPOR	Portugal	Southwestern Europe	Southern Portugal	Male
SP37	SPOR37	SPOR	Portugal	Southwestern Europe	Southern Portugal	Male
SP39	SPOR39	SPOR	Portugal	Southwestern Europe	Southern Portugal	Male
SP40	SPOR40	SPOR	Portugal	Southwestern Europe	Southern Portugal	Male
SP41	SPOR41	SPOR	Portugal	Southwestern Europe	Southern Portugal	Female
SP43	SPOR43	SPOR	Portugal	Southwestern Europe	Southern Portugal	Male
SP47	SPOR47	SPOR	Portugal	Southwestern Europe	Southern Portugal	Female
SP49	SPOR49	SPOR	Portugal	Southwestern Europe	Southern Portugal	Male
SP52	SPOR52	SPOR	Portugal	Southwestern Europe	Southern Portugal	Female
SP53	SPOR53	SPOR	Portugal	Southwestern Europe	Southern Portugal	Male
SP57	SPOR57	SPOR	Portugal	Southwestern Europe	Southern Portugal	Male
A002	MAAS002	MAAS	Morocco	Northwestern Africa	Morocco Berbers (Asni)	Male
A011	MAAS011	MAAS	Morocco	Northwestern Africa	Morocco Berbers (Asni)	Female
A038	MAAS038	MAAS	Morocco	Northwestern Africa	Morocco Berbers (Asni)	Female
A039	MAAS039	MAAS	Morocco	Northwestern Africa	Morocco Berbers (Asni)	Female
A048	MAAS048	MAAS	Morocco	Northwestern Africa	Morocco Berbers (Asni)	Female
A049	MAAS049	MAAS	Morocco	Northwestern Africa	Morocco Berbers (Asni)	Male
A068	MAAS068	MAAS	Morocco	Northwestern Africa	Morocco Berbers (Asni)	Female
A072	MAAS072	MAAS	Morocco	Northwestern Africa	Morocco Berbers (Asni)	Male
A074	MAAS074	MAAS	Morocco	Northwestern Africa	Morocco Berbers (Asni)	Male
A078	MAAS078	MAAS	Morocco	Northwestern Africa	Morocco Berbers (Asni)	Male
A089	MAAS089	MAAS	Morocco	Northwestern Africa	Morocco Berbers (Asni)	Female
A104	MAAS104	MAAS	Morocco	Northwestern Africa	Morocco Berbers (Asni)	Male
A110	MAAS110	MAAS	Morocco	Northwestern Africa	Morocco Berbers (Asni)	Female
A128	MAAS128	MAAS	Morocco	Northwestern Africa	Morocco Berbers (Asni)	Female
A129	MAAS129	MAAS	Morocco	Northwestern Africa	Morocco Berbers (Asni)	Male
B021	MABO021	MABO	Morocco	Northwestern Africa	Morocco Berbers (Bouhria)	Male
B033	MABO033	MABO	Morocco	Northwestern Africa	Morocco Berbers (Bouhria)	Male
B039	MABO039	MABO	Morocco	Northwestern Africa	Morocco Berbers (Bouhria)	Female
B049	MABO049	MABO	Morocco	Northwestern Africa	Morocco Berbers (Bouhria)	Male
B051	MABO051	MABO	Morocco	Northwestern Africa	Morocco Berbers (Bouhria)	Male
B053	MABO053	MABO	Morocco	Northwestern Africa	Morocco Berbers (Bouhria)	Female
B055	MABO055	MABO	Morocco	Northwestern Africa	Morocco Berbers (Bouhria)	Female
B092	MABO092	MABO	Morocco	Northwestern Africa	Morocco Berbers (Bouhria)	Female

Sample ID	Code	ACR	Country	Region	Population	Sex
B099	MABO099	MABO	Morocco	Northwestern Africa	Morocco Berbers (Bouhria)	Female
B100	MABO100	MABO	Morocco	Northwestern Africa	Morocco Berbers (Bouhria)	Female
B118	MABO118	MABO	Morocco	Northwestern Africa	Morocco Berbers (Bouhria)	Male
B131	MABO131	MABO	Morocco	Northwestern Africa	Morocco Berbers (Bouhria)	Female
F005	MAFI005	MAFI	Morocco	Northwestern Africa	Morocco Berbers (Figuig)	Male
F006	MAFI006	MAFI	Morocco	Northwestern Africa	Morocco Berbers (Figuig)	Male
F013	MAFI013	MAFI	Morocco	Northwestern Africa	Morocco Berbers (Figuig)	Female
F031	MAFI031	MAFI	Morocco	Northwestern Africa	Morocco Berbers (Figuig)	Female
F039	MAFI039	MAFI	Morocco	Northwestern Africa	Morocco Berbers (Figuig)	Female
F069	MAFI069	MAFI	Morocco	Northwestern Africa	Morocco Berbers (Figuig)	Female
F072	MAFI072	MAFI	Morocco	Northwestern Africa	Morocco Berbers (Figuig)	Female
F094	MAFI094	MAFI	Morocco	Northwestern Africa	Morocco Berbers (Figuig)	Male
F115	MAFI115	MAFI	Morocco	Northwestern Africa	Morocco Berbers (Figuig)	Male

Table S6. Mitochondrial DNA haplogroup (Hg) information in the samples analyzed in the present study. Hgs were assigned by combining information from previous analyses and the present work. In the latter case, Hgs were inferred by using Haplogrep tool (http://haplogrep.uibk.ac.at/). References: [1] Hernández et al. (2014) *BMC Genet* 15:11; [2] Hernández et al. (2015) *PLoS One* 10: e0139784; [3] Hernández et al. (2017) *BMC Genet* 18:46; [4] Coudray et al. (2009) *Ann Hum Genet* 73:196-214.

Sample ID	Code	Population	mtDNA Hg	Reference for mtDNA sequence
299532	SPWA299532	Western Andalusia (Huelva)	T	-
299590	SPWA299590	Western Andalusia (Huelva)	J1	-
299593	SPWA299593	Western Andalusia (Huelva)	U6d3a	1,2
299601	SPWA299601	Western Andalusia (Huelva)	U5b2a1	1
299605	SPWA299605	Western Andalusia (Huelva)	K2b1a1	-
334176	SPWA334176	Western Andalusia (Huelva)	J1c1	-
334183	SPWA334183	Western Andalusia (Huelva)	H4a1a4b1	1,3
334186	SPWA334186	Western Andalusia (Huelva)	J1	1
334200	SPWA334200	Western Andalusia (Huelva)	T	-
334206	SPWA334206	Western Andalusia (Huelva)	H3	1,3
334210	SPWA334210	Western Andalusia (Huelva)	J1	1
334211	SPWA334211	Western Andalusia (Huelva)	N	-
334230	SPWA334230	Western Andalusia (Huelva)	K	-
334240	SPWA334240	Western Andalusia (Huelva)	H6a1a7	1,3
334280	SPWA334280	Western Andalusia (Huelva)	H1	1,3
334282	SPWA334282	Western Andalusia (Huelva)	H1	1,3
335011	SPWA335011	Western Andalusia (Huelva)	K1a4a	1
335044	SPWA335044	Western Andalusia (Huelva)	H1	1,3
335066	SPWA335066	Western Andalusia (Huelva)	HV0	-
335069	SPWA335069	Western Andalusia (Huelva)	H1ah1	1,3
335077	SPWA335077	Western Andalusia (Huelva)	H6a1b	1,3
335081	SPWA335081	Western Andalusia (Huelva)	M1a1b1	1,2
335088	SPWA335088	Western Andalusia (Huelva)	U5b2a1	1
335097	SPWA335097	Western Andalusia (Huelva)	H6a1b	1,3
335114	SPWA335114	Western Andalusia (Huelva)	H18	1,3
601206	SPWA601206	Western Andalusia (Huelva)	T1a2	1
601215	SPWA601215	Western Andalusia (Huelva)	H1	1,3
601238	SPWA601238	Western Andalusia (Huelva)	H17	1,3
602790	SPWA602790	Western Andalusia (Huelva)	L2a1c6	1,2
602797	SPWA602797	Western Andalusia (Huelva)	HV0	1
602821	SPWA602821	Western Andalusia (Huelva)	T2	1
602822	SPWA602822	Western Andalusia (Huelva)	R0a2a	1
602825	SPWA602825	Western Andalusia (Huelva)	H1	1,3
602829	SPWA602829	Western Andalusia (Huelva)	K1a1b1c	-
602831	SPWA602831	Western Andalusia (Huelva)	K1a1b	-
930298	SPEA930298	Eastern Andalusia (Granada)	K1	-
930306	SPEA930306	Eastern Andalusia (Granada)	H1	1,3
930359	SPEA930359	Eastern Andalusia (Granada)	HV0a	-
930374	SPEA930374	Eastern Andalusia (Granada)	H20a	1,3
930382	SPEA930382	Eastern Andalusia (Granada)	H7	1,3
930400	SPEA930400	Eastern Andalusia (Granada)	H4a1	1,3
930401	SPEA930401	Eastern Andalusia (Granada)	U6b	1,2

Sample ID	Code	Population	mtDNA Hg	Reference for mtDNA sequence
932167	SPEA932167	Eastern Andalusia (Granada)	U5b	-
932176	SPEA932176	Eastern Andalusia (Granada)	H	1,3
932212	SPEA932212	Eastern Andalusia (Granada)	H2a2b	1,3
932232	SPEA932232	Eastern Andalusia (Granada)	HV0	1
932273	SPEA932273	Eastern Andalusia (Granada)	HV0a	1
932362	SPEA932362	Eastern Andalusia (Granada)	H5*	1,3
932396	SPEA932396	Eastern Andalusia (Granada)	N1b1	-
932404	SPEA932404	Eastern Andalusia (Granada)	H17	1,3
932406	SPEA932406	Eastern Andalusia (Granada)	J1	1
932408	SPEA932408	Eastern Andalusia (Granada)	H7	1,3
932431	SPEA932431	Eastern Andalusia (Granada)	HV0a	-
975465	SPEA975465	Eastern Andalusia (Granada)	U5a1d2a	-
975478	SPEA975478	Eastern Andalusia (Granada)	T	-
975512	SPEA975512	Eastern Andalusia (Granada)	H1	1,3
975546	SPEA975546	Eastern Andalusia (Granada)	H3*	1,3
975563	SPEA975563	Eastern Andalusia (Granada)	U4a1b1a	-
975568	SPEA975568	Eastern Andalusia (Granada)	H3*	1,3
975581	SPEA975581	Eastern Andalusia (Granada)	L3x2b1	1,2
975584	SPEA975584	Eastern Andalusia (Granada)	M1a2a1	1,2
975606	SPEA975606	Eastern Andalusia (Granada)	J1	-
988330	SPEA988330	Eastern Andalusia (Granada)	K2b1a1	-
988339	SPEA988339	Eastern Andalusia (Granada)	HV0a	-
988360	SPEA988360	Eastern Andalusia (Granada)	V+@72	-
988368	SPEA988368	Eastern Andalusia (Granada)	H13	1,3
988385	SPEA988385	Eastern Andalusia (Granada)	H1ba	1,3
988393	SPEA988393	Eastern Andalusia (Granada)	H4a1	1,3
988396	SPEA988396	Eastern Andalusia (Granada)	H1	1,3
988408	SPEA988408	Eastern Andalusia (Granada)	U5b	1
SP01	SPOR01	Southern Portugal	H	-
SP02	SPOR02	Southern Portugal	U5b	-
SP04	SPOR04	Southern Portugal	I	-
SP05	SPOR05	Southern Portugal	U6	-
SP06	SPOR06	Southern Portugal	H1	-
SP07	SPOR07	Southern Portugal	H1	-
SP08	SPOR08	Southern Portugal	J1b	-
SP09	SPOR09	Southern Portugal	H1b1d	-
SP11	SPOR11	Southern Portugal	J1	-
SP12	SPOR12	Southern Portugal	N	-
SP14	SPOR14	Southern Portugal	H1b1b	-
SP15	SPOR15	Southern Portugal	N	-
SP16	SPOR16	Southern Portugal	H	-
SP18	SPOR18	Southern Portugal	U5a1a1	-
SP19	SPOR19	Southern Portugal	H1	-
SP20	SPOR20	Southern Portugal	K1a4a	-
SP21	SPOR21	Southern Portugal	J1c1	-
SP23	SPOR23	Southern Portugal	J1c1	-
SP25	SPOR25	Southern Portugal	N	-
SP26	SPOR26	Southern Portugal	H1b1d	-
SP27	SPOR27	Southern Portugal	K1	-
SP28	SPOR28	Southern Portugal	N	-

Sample ID	Code	Population	mtDNA Hg	Reference for mtDNA sequence
SP29	SPOR29	Southern Portugal	H1c	-
SP31	SPOR31	Southern Portugal	H1	-
SP33	SPOR33	Southern Portugal	N	-
SP37	SPOR37	Southern Portugal	J1c1	-
SP39	SPOR39	Southern Portugal	H1c	-
SP40	SPOR40	Southern Portugal	H	-
SP41	SPOR41	Southern Portugal	K1	-
SP43	SPOR43	Southern Portugal	H1c	-
SP47	SPOR47	Southern Portugal	H1	-
SP49	SPOR49	Southern Portugal	J1c3c	-
SP52	SPOR52	Southern Portugal	H5'36	-
SP53	SPOR53	Southern Portugal	H3s	-
SP57	SPOR57	Southern Portugal	L2a1	-
SP150	SPOR150	Southern Portugal	H1	-
A002	MAAS002	Morocco Berbers (Asni)	L1b1a6	2,4
A011	MAAS011	Morocco Berbers (Asni)	HV0	-
A038	MAAS038	Morocco Berbers (Asni)	H1	-
A039	MAAS039	Morocco Berbers (Asni)	J2a	-
A048	MAAS048	Morocco Berbers (Asni)	L3e3'4'5	-
A049	MAAS049	Morocco Berbers (Asni)	U	2,4
A068	MAAS068	Morocco Berbers (Asni)	U6a3e	-
A072	MAAS072	Morocco Berbers (Asni)	M1a2a	2,4
A074	MAAS074	Morocco Berbers (Asni)	U6a3	2,4
A078	MAAS078	Morocco Berbers (Asni)	L3e5a	2,4
A089	MAAS089	Morocco Berbers (Asni)	U6a5c	-
A104	MAAS104	Morocco Berbers (Asni)	U6a3e	4
A110	MAAS110	Morocco Berbers (Asni)	N	-
A128	MAAS128	Morocco Berbers (Asni)	HV0a	-
A129	MAAS129	Morocco Berbers (Asni)	H	4
B021	MABO021	Morocco Berbers (Bouhria)	U6a8a	2,4
B033	MABO033	Morocco Berbers (Bouhria)	M1b2a	2,4
B039	MABO039	Morocco Berbers (Bouhria)	H	-
B049	MABO049	Morocco Berbers (Bouhria)	H4	4
B051	MABO051	Morocco Berbers (Bouhria)	H1	4
B053	MABO053	Morocco Berbers (Bouhria)	H	-
B055	MABO055	Morocco Berbers (Bouhria)	H1	-
B092	MABO092	Morocco Berbers (Bouhria)	H1	-
B099	MABO099	Morocco Berbers (Bouhria)	L1b	-
B100	MABO100	Morocco Berbers (Bouhria)	H1	-
B118	MABO118	Morocco Berbers (Bouhria)	U2e	4
B131	MABO131	Morocco Berbers (Bouhria)	H1	-
F005	MAFI005	Morocco Berbers (Figuig)	L1b1a8	2,4
F006	MAFI006	Morocco Berbers (Figuig)	L3b1a5	2,4
F013	MAFI013	Morocco Berbers (Figuig)	L3b1	2,4
F031	MAFI031	Morocco Berbers (Figuig)	J2a	4
F039	MAFI039	Morocco Berbers (Figuig)	L1b1a6	2,4
F069	MAFI069	Morocco Berbers (Figuig)	L2a1k	2,4
F072	MAFI072	Morocco Berbers (Figuig)	M1a1b	2,4
F094	MAFI094	Morocco Berbers (Figuig)	L3e3'4'5	4
F115	MAFI115	Morocco Berbers (Figuig)	L3b1a3	2,4

Table S7. High-resolution database built for genome-wide analysis with a density of 1,865,617 SNPs.

ACR	Country	Population	Region	N	Microarray	Reference
CEU	USA (CEPH)	CEPH	Europe	99	Whole genome	1000 Genomes phase 3 release
FIN	Finland	Finnish	North Europe	99	Whole genome	1000 Genomes phase 3 release
GBR	England and Scotland	British	Northwestern Europe	91	Whole genome	1000 Genomes phase 3 release
IBS	Spain	Spanish	Southwestern Europe	107	Whole genome	1000 Genomes phase 3 release
SPWA	*Spain*	*Western Andalusians (Huelva)*	*Southwestern Europe*	*35*	*Illumina Human Omni 2.5*	*present study*
SPEA	*Spain*	*Eastern Andalusians (Granada)*	*Southwestern Europe*	*35*	*Illumina Human Omni 2.5*	*present study*
SPOR	*Portugal*	*South Portugal*	*Southwestern Europe*	*36*	*Illumina Human Omni 2.5*	*present study*
TSI	Italy	Tuscan	Central Mediterranean Europe	107	Whole genome	1000 Genomes phase 3 release
MAAS	*Morocco*	*Asni*	*Northwestern Africa*	*15*	*Illumina Human Omni 2.5*	*present study*
MABO	*Morocco*	*Bouhria*	*Northwestern Africa*	*12*	*Illumina Human Omni 2.5*	*present study*
MAFI	*Morocco*	*Figuig*	*Northwestern Africa*	*9*	*Illumina Human Omni 2.5*	*present study*
GWD	Gambia	Mandinka	Western Africa	113	Whole genome	1000 Genomes phase 3 release
MSL	Sierra Leone	Mende	Western Africa	85	Whole genome	1000 Genomes phase 3 release
ESN	Nigeria	Esan	Central Africa	99	Whole genome	1000 Genomes phase 3 release
YRI	Nigeria	Yoruba	Central Africa	108	Whole genome	1000 Genomes phase 3 release

Table S8. Low-resolution database (84,586 SNPs).

ACR	Country	Population	Region	N	Microarray	Reference
CEU	USA (CEPH)	CEPH	Europe	99	Whole genome	1000 Genomes phase 3 release
FIN	Finland	Finnish	Northern Europe	99	Whole genome	1000 Genomes phase 3 release
GBR	England and Scotland	British	Northwestern Europe	91	Whole genome	1000 Genomes phase 3 release
FRFR	France	French	Western Europe	28	Illumina 650K	HGDP-CEPH / Li et al., 2008
FRBA	France	French Basque	Western Europe	24	Illumina 650K	HGDP-CEPH / Li et al., 2008
IBS	Spain	Spanish	Southwestern Europe	107	Whole genome	1000 Genomes phase 3 release
SPGA	Spain	Galicia	Southwestern Europe	17	Affymetrix 6.0	Botigué et al., 2013
SPBA	Spain	Basques	Southwestern Europe	20	Affymetrix 6.0	Henn et al., 2012
SPAN	Spain	Andalusia	Southwestern Europe	17	Affymetrix 6.0	Botigué et al., 2013
SPWA	*Spain*	*Western Andalusians (Huelva)*	*Southwestern Europe*	*35*	*Illumina Human Omni 2.5*	*present study*
SPEA	*Spain*	*Eastern Andalusians (Granada)*	*Southwestern Europe*	*35*	*Illumina Human Omni 2.5*	*present study*
SPOR	*Portugal*	*South Portugal*	*Southwestern Europe*	*36*	*Illumina Human Omni 2.5*	*present study*
TSI	Italy	Tuscan	Central Mediterranean Europe	107	Whole genome	1000 Genomes phase 3 release
ITNO	Italy	North Italian (Bergamo)	Central Mediterranean Europe	12	Illumina 650K	HGDP-CEPH / Li et al., 2008
ISBE	Israel	Bedouin (Negev)	Near East	46	Illumina 650K	HGDP-CEPH / Li et al., 2008
ISDR	Israel	Druze (Carmel)	Near East	42	Illumina 650K	HGDP-CEPH / Li et al., 2008
ISPA	Israel	Palestinian (Central)	Near East	46	Illumina 650K	HGDP-CEPH / Li et al., 2008
MAAS	*Morocco*	*Asni*	*Northwestern Africa*	*15*	*Illumina Human Omni 2.5*	*present study*
MABO	*Morocco*	*Bouhria*	*Northwestern Africa*	*12*	*Illumina Human Omni 2.5*	*present study*
MAFI	*Morocco*	*Figuig*	*Northwestern Africa*	*9*	*Illumina Human Omni 2.5*	*present study*
MANO	Morocco	Morocco North	Northwestern Africa	18	Affymetrix 6.0	Henn et al., 2012
MASO	Morocco	Morocco South	Northwestern Africa	16	Affymetrix 6.0	Henn et al., 2012
DZMO	Algeria	Mozabite	Northwestern Africa	29	Illumina 650K	HGDP-CEPH / Li et al., 2008
ALG	Algeria	Alger	Northwestern Africa	19	Affymetrix 6.0	Henn et al., 2012

ACR	Country	Population	Region	N	Microarray	Reference
TUN	Tunisia	Tunisian Berbers (Chenini)	Northwestern Africa	18	Affymetrix 6.0	Henn et al., 2012
LIB	Libya	Libya	Northeastern Africa	17	Affymetrix 6.0	Henn et al., 2012
EGY	Egypt	Egypt	Northeastern Africa	19	Affymetrix 6.0	Henn et al., 2012
WSA	Western Sahara	Saharawi	Western Africa	18	Affymetrix 6.0	Henn et al., 2012
SNMA	Senegal	Mandenka	Western Africa	22	Illumina 650K	HGDP-CEPH / Li et al., 2008
GWD	Gambia	Mandinka	Western Africa	113	Whole genome	1000 Genomes phase 3 release
MSL	Sierra Leone	Mende	Western Africa	85	Whole genome	1000 Genomes phase 3 release
BFGM	Burkina Faso	Gurmantche	Western Africa	15	Illumina Human Omni 2.5	Triska et al., 2015
BFGR	Burkina Faso	Gurunsi	Western Africa	16	Illumina Human Omni 2.5	Triska et al., 2015
BFMO	Burkina Faso	Mossi	Western Africa	17	Illumina Human Omni 2.5	Triska et al., 2015
ESN	Nigeria	Esan	Central Africa	99	Whole genome	1000 Genomes phase 3 release
YRI	Nigeria	Yoruba	Central Africa	108	Whole genome	1000 Genomes phase 3 release

References

Botigué et al. 2013. Gene flow from North Africa contributes to differential human genetic diversity in southern Europe. *Proc Natl Acad Sci USA* 110:11791–6.
Henn et al. 2012. Genomic ancestry of North Africans supports back-to-Africa migrations. *PLoS Genet.* 8:e1002397.
Li et al. 2008. Worldwide Human Relationships Inferred from Genome-Wide Patterns of Variation. *Science* 319:1100–1104.
Triska et al. 2015. Extensive admixture and selective pressure across the Sahel Belt. *Genome Biol Evol* 7:3484–3495.

Table S9. Scenarios tested for different combinations of recipient/donor clusters as defined in the initial painting protocol of fineSTRUCTURE. See details on clusters in **Table S3**.

	TEST A				TEST B	
	Recipient		Donors		Donors	
IBERIA	C23WAND	Western Andalusia	C4	Near East	C4	Near East
	C24IBE1	Iberia	C14	North Africa (Maghreb)	C10	North Africa (Morocco)
	C25AND	Andalusia	C21	Europe	C21	Europe
	C26IBE2	Iberia	C41	Sub-Saharan Africa	C41	Sub-Saharan Africa
	C29IBE3	Western Iberia				

	TEST A				TEST C		TEST D		TEST E	
	Recipient		Donors		Donors		Donors		Donors	
NORTH AFRICA	C10MOR	Morocco	C4	Near East	C4	Near East	C4	Near East	C4	Near East
	C11WSA	Western Sahara	C22	Central Mediterranean Europe	C22	Central Mediterranean Europe	C22	Central Mediterranean Europe	C22	Central Mediterranean Europe
	C13MAGH1	Morocco & Algeria	C25	Western Mediterranean Europe (Andalusia)	C23	Western Mediterranean Europe (W Andalusia)	C24	Western Mediterranean Europe (general Iberia)	C29	Western Mediterranean Europe (W Iberia)
	C14MAGH2	Morocco & Algeria	C41	Sub-Saharan Africa	C41	Sub-Saharan Africa	C41	Sub-Saharan Africa	C41	Sub-Saharan Africa
	C16NAFR	North Africa (longitudinal)								

Table S10. Details on aDNA samples selected for the longitudinal study of genomic variation in the western Mediterranean.

Sample ID	Population	Period	Area	Accession Number	Repository	Reference
TOR.6	El Toro, Andalusia	Early Neolithic	Spain	ERR2131415	European Nucleotide Archive (ENA)	Fregel et al. 2018
TOR.7	El Toro, Andalusia	Early Neolithic	Spain	ERR2131416	European Nucleotide Archive (ENA)	Fregel et al. 2018
TOR.8	El Toro, Andalusia	Early Neolithic	Spain	ERR2131417	European Nucleotide Archive (ENA)	Fregel et al. 2018
TOR.11	El Toro, Andalusia	Early Neolithic	Spain	ERR2131418	European Nucleotide Archive (ENA)	Fregel et al. 2018
MUR	Murciélagos de Zuheros, Andalusia	Early Neolithic	Spain	ERS2059524	European Nucleotide Archive (ENA)	Valdiosera et al. 2018
C40331	Cueva de los Cuarenta, Andalusia	Late Neolithic/Copper Age	Spain	ERS2059522	European Nucleotide Archive (ENA)	Valdiosera et al. 2018
PIR001	Priego de Córdoba, Andalusia	Early Bronze Age	Spain	ERS2059525	European Nucleotide Archive (ENA)	Valdiosera et al. 2018
LC41	Lugar do Canto	Middle Neolithic	Portugal	ERS1240044	European Nucleotide Archive (ENA)	Martiniano et al. 2017
LC42	Lugar do Canto	Middle Neolithic	Portugal	ERS1240045	European Nucleotide Archive (ENA)	Martiniano et al. 2017
LC44	Lugar do Canto	Middle Neolithic	Portugal	ERS1240046	European Nucleotide Archive (ENA)	Martiniano et al. 2017
LC45	Lugar do Canto	Middle Neolithic	Portugal	ERS1240047	European Nucleotide Archive (ENA)	Martiniano et al. 2017
CA117B	Cabeco da Arruda	Late Neolithic	Portugal	ERS1240048	European Nucleotide Archive (ENA)	Martiniano et al. 2017
CA122A	Cabeco da Arruda	Late Neolithic	Portugal	ERS1240049	European Nucleotide Archive (ENA)	Martiniano et al. 2017
CM364	Cova da Moura	Late Neolithic	Portugal	ERS1240050	European Nucleotide Archive (ENA)	Martiniano et al. 2017
CM9B	Cova da Moura	Late Neolithic	Portugal	ERS1240051	European Nucleotide Archive (ENA)	Martiniano et al. 2017
DA96B	Dolmen de Ansiao	Late Neolithic	Portugal	ERS1240052	European Nucleotide Archive (ENA)	Martiniano et al. 2017
MC337A	Monte Canelas 1	Late Neolithic	Portugal	ERS1240053	European Nucleotide Archive (ENA)	Martiniano et al. 2017
MG104	Monte do Gato	Middle Bronze Age	Portugal	ERS1240054	European Nucleotide Archive (ENA)	Martiniano et al. 2017
TV32032	Torre Velha 3	Middle Bronze Age	Portugal	ERS1240055	European Nucleotide Archive (ENA)	Martiniano et al. 2017
TV3831	Torre Velha 3	Middle Bronze Age	Portugal	ERS1240056	European Nucleotide Archive (ENA)	Martiniano et al. 2017
VO10207	Monte Vale do Ouro	Middle Bronze Age	Portugal	ERS1240057	European Nucleotide Archive (ENA)	Martiniano et al. 2017
TAF011	Taforalt (Grotte des Pigeons)	Late Stone Age (LSA)	Morocco	SRR6664775	Sequence Read Archive (NCBI)	van de Loosdrecht et al. 2018
TAF013	Taforalt (Grotte des Pigeons)	Late Stone Age (LSA)	Morocco	SRR6664773	Sequence Read Archive (NCBI)	van de Loosdrecht et al. 2018
TAF010	Taforalt (Grotte des Pigeons)	Late Stone Age (LSA)	Morocco	SRR6664774	Sequence Read Archive (NCBI)	van de Loosdrecht et al. 2018
TAF012	Taforalt (Grotte des Pigeons)	Late Stone Age (LSA)	Morocco	SRR6664772	Sequence Read Archive (NCBI)	van de Loosdrecht et al. 2018
TAF014	Taforalt (Grotte des Pigeons)	Late Stone Age (LSA)	Morocco	SRR6664770	Sequence Read Archive (NCBI)	van de Loosdrecht et al. 2018
IAM.3	Ifri n'Amr or Moussa (IAM)	Early Neolithic	Morocco	ERR2131406	European Nucleotide Archive (ENA)	Fregel et al. 2018
IAM.4	Ifri n'Amr or Moussa (IAM)	Early Neolithic	Morocco	ERR2131407	European Nucleotide Archive (ENA)	Fregel et al. 2018
IAM.5	Ifri n'Amr or Moussa (IAM)	Early Neolithic	Morocco	ERR2131408	European Nucleotide Archive (ENA)	Fregel et al. 2018

Sample ID	Population	Period	Area	Accession Number	Repository	Reference
IAM.6	Ifri n'Amr or Moussa (IAM)	Early Neolithic	Morocco	ERR2131409	European Nucleotide Archive (ENA)	Fregel et al. 2018
IAM.7	Ifri n'Amr or Moussa (IAM)	Early Neolithic	Morocco	ERR2131410	European Nucleotide Archive (ENA)	Fregel et al. 2018
KEB.1	Kelifel Boroud (KEB)	Late Neolithic	Morocco	ERR2131411	European Nucleotide Archive (ENA)	Fregel et al. 2018
KEB.4	Kelifel Boroud (KEB)	Late Neolithic	Morocco	ERR2131412	European Nucleotide Archive (ENA)	Fregel et al. 2018
KEB.6	Kelifel Boroud (KEB)	Late Neolithic	Morocco	ERR2131413	European Nucleotide Archive (ENA)	Fregel et al. 2018
KEB.8	Kelifel Boroud (KEB)	Late Neolithic	Morocco	ERR2131414	European Nucleotide Archive (ENA)	Fregel et al. 2018

References

Fregel R et al. 2018. Ancient genomes from North Africa evidence prehistoric migrations to the Maghreb from both the Levant and Europe. *Proc Natl Acad Sci USA* 115:6774–6779.

Martiniano R et al. 2017. The population genomics of archaeological transition in west Iberia: Investigation of ancient substructure using imputation and haplotype-based methods. *PLoS Genet.* 13:1–24.

Valdiosera C et al. 2018. Four millennia of Iberian biomolecular prehistory illustrate the impact of prehistoric migrations at the far end of Eurasia. *Proc Natl Acad Sci.* 115:3428–3433.

van de Loosdrecht et al. 2018. Pleistocene North African genomes link Near Eastern and sub-Saharan African human populations. *Science* 360:548–552.
